# Supplementary figures and images for: KSR2 Mutations Are Associated with Obesity, Insulin Resistance, and Impaired Cellular Fuel Oxidation
Source: Cell. 2013 Nov 7;155(4):765–77. doi: 10.1016/j.cell.2013.09.058 (PMC3898740; doi:10.1016/j.cell.2013.09.058)

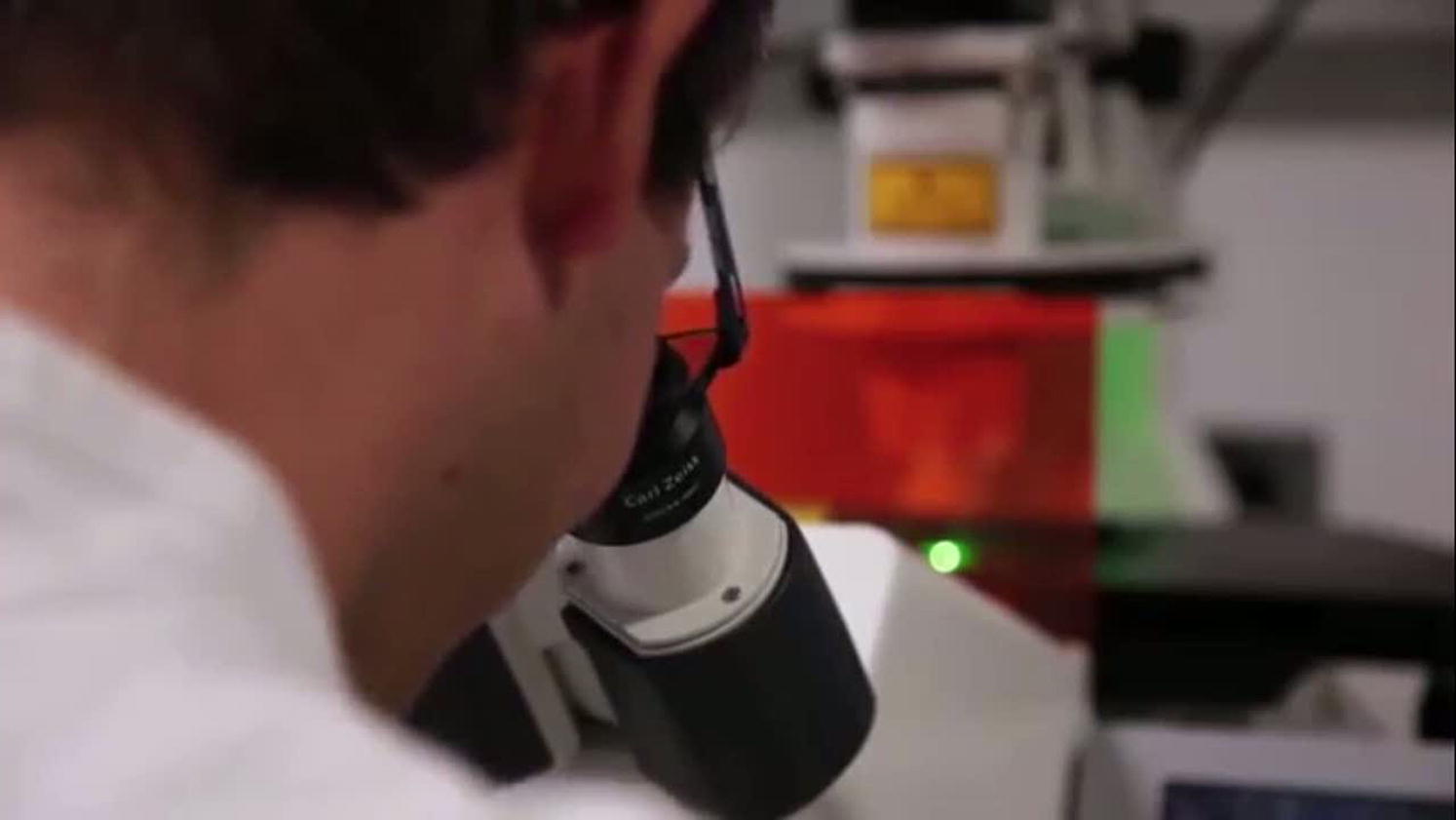

Supplement: Supplementary file 1 [file mmc2.jpg]
