# Supplementary material for: KSR2 Mutations Are Associated with Obesity, Insulin Resistance, and Impaired Cellular Fuel Oxidation
Source: Cell. 2013 Nov 7;155(4):765–77. doi: 10.1016/j.cell.2013.09.058 (PMC3898740; doi:10.1016/j.cell.2013.09.058)
Supplement: Document S1. Tables S1–S4 [file mmc1.pdf]

Table S1. Cosegregation of *KSR2* Mutations with Overweight/Obesity in Family Members, Related to Figure 1

		Heterozygous family members	Wild type family members	Prevalence in population based cohort/publically available databases		
**Mutation**	**Patients**	**BMI for adults; BMI SDS for children**	**BMI for adults; BMI SDS for children**	**Ely Study**	**dbSNP**	**MAF NHLBI Exomes**
K62N	1		41; 0	-	-	-
F89L	1		34, 18	-	-	-
W95R	1			-	-	-
D124Y	1	44		-	-	0.008
L148V	1			-	-	-
T175R	8			5	rs200451365	0.123
T215A	1			-	-	-
R246S	-			1	-	-
R253W#	1	32, 28	29	-	-	-
D323E	10			5	rs141457085	0.2528
A373T	1		40	-	-	0.0159
R397H	1			-	-	R397C (0.0158)
V511Cfsx29	1			-	-	-
P542L	1		48, 33, 32	-	-	-
Y569X	1	28	28	-	-	-
E620D	1			-	-	-
P662L*	1	38	25	-	-	-
E667V*	1		24	-	rs150163296	0.00713
R684C	1			-	-	-
R745W	-			1	-	0.0083
I801L	1			-	-	-
F807Qfsx41*	1	27; 0.4, 0.6	29	-	-	-
G816D	1	27	28, 37, 32	1	-	-
R818Q*	1	42, 45, 31	40, 31; 2.7, 1.2	-	-	R818W (0.008)
L822Pfsx26	1			-	-	-
R823H	1			-	rs183487509	0.0079
R838H	-			1	-	0.008
D843N*	1	30	26	-	-	0.008
S904L*	4	28, 37 ,27; 1.2	35, 32; 0.7	2	rs201637020	0.1827
R936L*	1	34; 2.4	28	-	-	-

Where consent was given, family members of probands carrying *KSR2* mutations were genotyped. The BMI (kg/m^2) (or BMI sds in children) of 19 family members who were heterozygous for variants or 25 family members who were wild-type, is noted (# denotes homozygous carriers in one family). Some individuals consented to take part in detailed physiological studies and are indicated (*). Additional data is shown to compare the prevalence of *KSR2* mutations found in severely obese individuals with those found in controls from a population based cohort (the Ely Study) and publically available databases; MAF = minor allele frequency.

Table S2. Permutation Analysis of Variants Found in Cases and Controls, Related to Figure 1

ORIGINAL RESULTS

Filter		cases		controls		OR	p-value
		# with variants	total	# with variants	total		
MAF < 0.005		45	2101	16	1536	2.08	0.0124
MAF < 0.001		28	2101	6	1536	3.44	0.0029
MAF < 0.0005		24	2101	4	1536	4.43	0.0019
**Permutation p-value**							**0.0025**

REPLICATION RESULTS

Filter	cases		controls		OR	p-value
	# with variants	total	# with variants	total		
MAF < 0.005	6	238	26	1117	1.09	0.8153
MAF < 0.001	4	238	10	1117	1.89	0.2864
MAF < 0.0005	4	238	8	1117	2.37	0.2416
**Permutation p-value**						**0.9987**

COMBINED RESULTS

Filter		cases		controls		OR	p-value
		# with variants	total	# with variants	total		
MAF < 0.005		73	2339	67	2653	1.24	0.2291
MAF < 0.001		34	2339	29	2653	1.33	0.3094
MAF < 0.0005		29	2339	13	2653	2.55	0.0047
**Permutation p-value**							**0.0063**

MAF = minor allele frequency; OR = odds ratio; # number.

Table S3. Metabolic Parameters in *KSR2* Mutation Carriers and Obese Controls, Related to Figure 4

	KSR2	**Controls**
Systolic Blood Pressure (mmHg)	130.2 ± 3.8	129.1 ± 2.3
Diastolic Blood Pressure (mmHg)	75.3 ± 3.1	78.9 ± 1.5
Total Cholesterol (mmol/l)	4.7 ± 0.2	4.9 ± 0.2
LDL-Cholesterol (mmol/l)	3.0 ± 0.1	3.2 ± 0.1
HDL-Cholesterol (mmol/l)	1.1 ± 0.1	1.1 ± 0.1
Triglycerides (mmol/l)	1.2 ± 0.1	1.4 ± 0.2
Adiponectin (mg/l)	6.4 (range 3.3 to 12.8)	7.7 (range 0.82 to 28.5)
Urinary Norepinephrine (nmol/24hr)	109 ± 5	Reference range: 63 - 471
Urinary Epinephrine (nmol/24hr)	23 ± 1	Reference range: 4 - 127
Urinary Dopamine (nmol/l)	1149 ± 34	Reference range: 70 - 1900
Thyroid stimulating hormone (mU/L)	1.9 ± 0.1	Reference range: 0.35 - 5.5
Free thyroxine (pmol/l)	14 ± 0.4	Reference range: 11.5 - 22.7

Means ±SEM are shown. Where parameters are altered in obesity, data from obese controls is provided. Otherwise, laboratory reference ranges are noted.

Table S4. Body Composition Data for *Ksr2*$^{-/-}$ Mice and Their *Ksr2*$^{+/+}$ Littermates, Related to Figure 5

Mice	N	Age	Body Weight (g)	Body Fat (g)	LBM (g)	% Body Fat
**Male**						
Ksr2$^{-/-}$ ad lib	4	5 weeks	23 ± 0.3	6 ± 0.3 **	17 ± 0.3	28 ± 0.9 **
Ksr2$^{-/-}$ PF	9	5 weeks	22 ± 1.5	6 ± 0.7 **	16 ± 0.8	25 ± 1.5 ***
Ksr2$^{+/+}$	12	5 weeks	21 ± 0.5	3 ± 0.2	17 ± 0.4	16 ± 0.8
Ksr2$^{-/-}$ ad lib	4	9 weeks	48 ± 1.2 ^^	22 ± 1 ^^	26 ± 0.3 ^^	46 ± 1 ^^
Ksr2$^{-/-}$ PF	9	9 weeks	28 ± 1.1	10 ± 0.8 ***	18 ± 0.4 *	35 ± 1.3 ***
Ksr2$^{+/+}$	12	9 weeks	26 ± 0.8	6 ± 0.5	20 ± 0.6	22 ± 1.5
Ksr2$^{-/-}$ PF	9	18 weeks	37 ± 1.3 **	16 ± 0.9 ***	22 ± 1.3	41 ± 0.4 ***
Ksr2$^{+/+}$	12	18 weeks	31 ± 1.6	8 ± 1	23 ± 2.2	24 ± 0.7
**Female**						
Ksr2$^{-/-}$ ad lib	3	5 weeks	18 ± 2.1	5 ± 1	13 ± 1.2	26 ± 3.1
Ksr2$^{-/-}$ PF	6	5 weeks	18 ± 1.4	5 ± 0.7	13 ± 0.6	26 ± 2.2 *
Ksr2$^{+/+}$	10	5 weeks	17 ± 0.2	3 ± 0.2	13 ± 0.2	19 ± 0.9
Ksr2$^{-/-}$ ad lib	3	9 weeks	40 ± 5.3 ^^	20 ± 3.3 ^^	20 ± 2 ^	50 ± 1.9 ^
Ksr2$^{-/-}$ PF	6	9 weeks	22 ± 0.8	8 ± 0.5	15 ± 0.4	34 ± 1.4 **
Ksr2$^{+/+}$	10	9 weeks	20 ± 0.6	5 ± 0.5	15 ± 0.4	24 ± 2.1
Ksr2$^{-/-}$ ad lib	1	18 weeks	49	24	25	49
Ksr2$^{-/-}$ PF	6	18 weeks	27 ± 0.7 *	10 ± 0.9 ***	17 ± 0.4	37 ± 2.7 ***
Ksr2$^{+/+}$	10	18 weeks	23 ± 0.8	5 ± 0.6	18 ± 0.5	22 ± 1.8

LBM, lean body mass; PF, pair-fed. Different from *Ksr2*$^{+/+}$: * $p < 0.05$, ** $p < 0.01$, *** $p < 0.001$; *Ksr2*$^{-/-}$ ad lib different from *Ksr2*$^{-/-}$ PF and *Ksr2*$^{+/+}$: ^ $p < 0.01$, ^^ $p < 0.001$.
