# Supplementary material for: KSR2 Mutations Are Associated with Obesity, Insulin Resistance, and Impaired Cellular Fuel Oxidation
Source: Cell. 2013 Nov 7;155(4):765–77. doi: 10.1016/j.cell.2013.09.058 (PMC3898740; doi:10.1016/j.cell.2013.09.058)
Supplement: Document S2. Article plus Supplemental Information [file mmc3.pdf]

Cell

KSR2 Mutations Are Associated with Obesity, Insulin Resistance, and Impaired Cellular Fuel Oxidation

Laura R. Pearce,[1,9] Neli Atanassova,[1,9] Matthew C. Banton,[1,9] Bill Bottomley,[2] Agatha A. van der Klaauw,[1] Jean-Pierre Revelli,[3] Audrey Hendricks,[2] Julia M. Keogh,[1] Elana Henning,[1] Deon Doree,[3] Sabrina Jeter-Jones,[3] Sumedha Garg,[1] Elena G. Bochukova,[1] Rebecca Bounds,[1] Sofie Ashford,[1] Emma Gayton,[2] Peter C. Hindmarsh,[4] Julian P.H. Shield,[5] Elizabeth Crowne,[5] David Barford,[6] Nick J. Wareham,[7] UK10K consortium,[2] Stephen O'Rahilly,[1] Michael P. Murphy,[8] David R. Powell,[3] Ines Barroso,[1,2] and I. Sadaf Farooqi[1,*]
[1]University of Cambridge Metabolic Research Laboratories and NIHR Cambridge Biomedical Research Centre, Wellcome Trust-MRC Institute of Metabolic Science, Addenbrooke's Hospital, Cambridge CB2 0QQ, UK
[2]Wellcome Trust Sanger Institute, Cambridge, CB10 1SA, UK
[3]Lexicon Pharmaceuticals, The Woodlands, TX 77381, USA
[4]Institute of Child Health, University College London, London WC1E 6BT, UK
[5]University of Bristol and Bristol Royal Hospital for Children, Bristol BS2 8BJ, UK
[6]Institute of Cancer Research, Chester Beatty Laboratories, London SW3 6JB, UK
[7]MRC Epidemiology Unit, Wellcome Trust-MRC Institute of Metabolic Science, Addenbrooke's Hospital, Cambridge CB2 0QQ, UK
[8]MRC Mitochondrial Biology Unit, Wellcome Trust/MRC Building, Hills Road, Cambridge CB2 0XY, UK
[9]These authors contributed equally to this work
*Correspondence: isf20@cam.ac.uk
http://dx.doi.org/10.1016/j.cell.2013.09.058
This is an open-access article distributed under the terms of the Creative Commons Attribution License, which permits unrestricted use, distribution, and reproduction in any medium, provided the original author and source are credited.

SUMMARY

Kinase suppressor of Ras 2 (KSR2) is an intracellular scaffolding protein involved in multiple signaling pathways. Targeted deletion of *Ksr2* leads to obesity in mice, suggesting a role in energy homeostasis. We explored the role of *KSR2* in humans by sequencing 2,101 individuals with severe early-onset obesity and 1,536 controls. We identified multiple rare variants in *KSR2* that disrupt signaling through the Raf-MEK-ERK pathway and impair cellular fatty acid oxidation and glucose oxidation in transfected cells; effects that can be ameliorated by the commonly prescribed antidiabetic drug, metformin. Mutation carriers exhibit hyperphagia in childhood, low heart rate, reduced basal metabolic rate and severe insulin resistance. These data establish KSR2 as an important regulator of energy intake, energy expenditure, and substrate utilization in humans. Modulation of KSR2-mediated effects may represent a novel therapeutic strategy for obesity and type 2 diabetes.

INTRODUCTION

Cells sense the nutritional status of the organism, monitor intracellular energy stores, and transmit this information to signal transduction pathways that drive cellular proliferation and differentiation. The Ras-Raf-MEK signaling pathway is fundamental to cellular metabolism and growth in humans; inherited mutations involving this pathway cause disorders of early growth and development (Tartaglia and Gelb, 2010), whereas sporadic activating mutations have been identified in at least 30% of human cancers (Lawrence et al., 2008). Circulating hormones and growth factors activate cell surface receptor tyrosine kinases, stimulating the GTP loading of Ras, which permits recruitment of the cytosolic Ser/Thr protein kinase Raf to the plasma membrane, where it is activated (McKay and Morrison, 2007). Membrane-bound Raf phosphorylates and activates the dual-specificity kinase MEK, which in turn phosphorylates ERK, which then translocates to the nucleus where it regulates gene expression.

The kinase suppressors of Ras proteins (KSR1 and KSR2) were originally identified from genetic screens in *Drosophila* and *C. elegans* (Kornfeld et al., 1995; Sundaram and Han, 1995; Therrien et al., 1995) and found to act as positive regulators of the Ras-Raf-MEK signaling pathway by acting as scaffolding proteins (Nguyen et al., 2002). Both KSR1 and 2 bind to Raf, MEK, and ERK, facilitating their phosphorylation and activation (Denouel-Galy et al., 1998; Dougherty et al., 2009; Roy et al., 2002; Therrien et al., 1996). In addition, KSR proteins translocate to the plasma membrane in response to growth factor stimulation (Müller et al., 2001), thereby regulating the spatial and temporal dynamics of Ras-Raf-MEK signaling as well as increasing the efficiency and specificity of this pathway. The recently solved KSR2-MEK1 structure has revealed how KSR2 regulates MEK activation by interacting with B-Raf (Brennan et al., 2011). Proteomic studies have also shown that KSR2 interacts with multiple proteins including AMP-activated protein kinase (AMPK) (Costanzo-Garvey et al., 2009; Liu et al., 2009), a phylogenetically conserved Ser/Thr

A

Mutation	Nucleotide Change	State	Patients	Controls	BMI (sds) of patients upon recruitment	BMI of controls upon recruitment	PolyPhen2	SIFT
K62N*	c.186G>C	Het	1	-	30.4 (5.8)	-	Probably damaging	Damaging
F89L*	c.265T>C	Het	1	-	24.2 (4.0)	-	Probably damaging	Damaging
W95R	c.283T>C	Het	1	-	45.3 (4.1)	-	Probably damaging	Damaging
D124Y*	c.370G>T	Het	1	-	79.1 (4.8)	-	Probably damaging	Damaging
L148V	c.442C>G	Het	1	-	32.8 (5.6)	-	Benign	Damaging
T175R	c.524C>G	Het	8	5	29.6 (4.9), 24.6 (3.8), 56.9 (4.6), 41.8 (5.4), 35.9 (3.3), 43.3 (3.9), 28 (4.5), 30 (5.0)	25.8, 25, 29.6, 26, 25.5	Benign	Tolerated
T215A	c.643A>G	Het	1	-	28.9 (5.0)	-	Benign	Tolerated
R246S	c.736C>A	Het	-	1	-	27	Benign	Tolerated
R253W*	c.575C>T	Hom	1	-	33.1 (3.8)	-	Probably damaging	Damaging
D323E	c.969C>G	Het (Hom)	9 (1)	5	33.1 (3.8), 31.9 (3.2), 44.8 (3.9), 22.3 (3.5), 41.0 (3.7), 52.3 (4.2), 24.1 (3.7), 40.7 (3.7), 27.2 (4.3), 29 (4.0)	28, 26 , 25.8, 25.8, 28.6	Benign	Tolerated
A373T	c.1117G>A	Het	1	-	27.9 (4.8)	-	Benign	Tolerated
R397H	c.1190G>A	Het	1	-	26.1 (4.2)	-	Benign	Tolerated
V511Cfsx29	c.1527insT	Het	1	-	26.8 (4.5)	-	Damaging	Damaging
P542L	c.1625C>T	Het	1	-	38.4 (4.7)	-	Probably damaging	Tolerated
R554Q	c.1661G>A	Het (Hom)	20 (2)	25	Het: 17.1 (1.0), 33.3 (2.9), 22.3 (2.8), 36.2 (3.4), 27.0 (4.1), 31.1 (4.4), 27.8 (2.9), 27.2 (4.2), 38.1 (3.6), 24.0 (3.0), 34.6 (3.2), 38.6 (3.4), 30.6 (3.3), 26.9 (4.6), 54.0 (4.5), 28.7 (5.3), 46.0 (3.9), 26.7 (4.3), 54.7 (4.5), 0 (). Hom: 31.3 (5.3), 27.8 (3.0)	25.6,25.3, 22.4, 22.2, 28, 19.7, 31, 27.7, 21.5, 28, 28, 20.4, 30.3, 22.3, 20.2, 22.9, 25.2, 30, 20.2, 24.3 45.8, 21.5, 25.9, 23.9, no data	Benign	Tolerated
Y569X*	c.1707C>G	Het	1	-	23.6 (3.1)	-	Damaging	Damaging
E620D	c.1860G>C	Het	1	-	25.5 (1.6)	-	Benign	Tolerated
P662L	c.1985C>T	Het	1	-	29.2 (5.4)	-	Probably damaging	Damaging
E667V	c.2000A>T	Het	1	-	26.2 (4.5)	-	Benign	Damaging
R684C	c.2050C>T	Het	1	-	42.3 (3.8)	-	Probably damaging	Damaging
R745W	c.2233C>T	Het	-	1	-	34	Probably damaging	Damaging
I801L	c.2401A>C	Het	1	-	48.7 (4.2)	-	Probably damaging	Damaging
F807Qfsx41	c.2419-2420delTT	Het	1	-	24.6 (4.3)	-	Damaging	Damaging
G816D	c.2447G>A	Het	1	1	33.2 (3.3)	29	Possibly damaging	Tolerated
R818Q*	c.2453G>A	Het	1	-	27.9 (3.6)	-	Probably damaging	Tolerated
L822Pfsx26	c.2465-2466delTG	Het	1	-	45.0 (3.9)	-	Damaging	Damaging
R823H	c.2468G>A	Het	1	-	27.7 (2.9)	-	Probably damaging	Damaging
R838H	c.2513G>A	Het	-	1	-	27	Benign	Damaging
D843N	c.2527G>A	Het	1	-	28.4 (3.1)	-	Possibly damaging	Tolerated
S904L	c.2711C>T	Het	4	2	30.6 (3.1), 36.2 (6.6), 25.1 (4.2), 25.4 (4.6)	36, 21	Benign	Damaging
R936L	c.2807G>T	Het	1	-	30.4 (3.2)	-	Probably damaging	Damaging

B

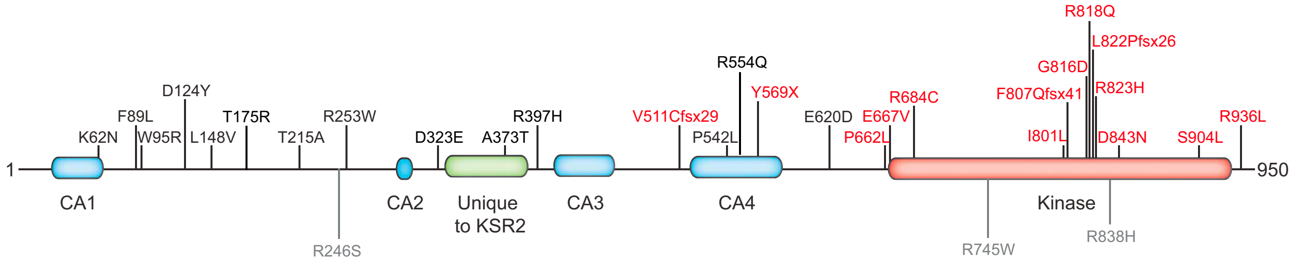

Figure 1. Identification of Multiple *KSR2* Mutations in Severely Obese Individuals and Controls

(A) Mutations in *KSR2* identified in individuals with severe early-onset obesity and in controls (shown in gray). Mutations identified by whole-exome sequencing are marked with an asterisk; all others were identified by Sanger sequencing. Mutation numbering is based on Ensembl transcript ID ENST00000339824 and Protein ID ENSP00000339952; Het = heterozygous and Hom = homozygous. As BMI (kg/m^2) varies with age and gender, the BMI and BMI SD scores (sds) are noted. The potential functional consequences of each mutation were assessed using Polyphen2 and SIFT.

(legend continued on next page)

protein kinase that acts as a fuel sensor, monitoring cellular energy status in eukaryotes. Under conditions of nutrient deprivation and cellular stress, intracellular ATP levels fall and levels of AMP rise, promoting AMPK activation that in turn promotes catabolic processes and inhibits anabolic pathways (Hardie et al., 2012). The interaction between KSR2 and AMPK has been suggested to underpin some of the abnormalities of energy homeostasis and metabolism seen in *Ksr2*$^{-/-}$ mice, which include obesity, high insulin levels, and impaired glucose tolerance (Brommage et al., 2008; Costanzo-Garvey et al., 2009; Revelli et al., 2011). *Ksr2*$^{+/-}$ mice also develop obesity when fed on a high-fat diet, indicating that the physiological effects of *Ksr2* disruption on energy balance are dosage dependent (Revelli et al., 2011).

In this study, we found a large number of variants in *KSR2* in individuals with severe early-onset obesity. Many of the variants studied impaired signaling through the Raf-MEK-ERK pathway. While some of the variants reduced the interaction between KSR2 and AMPK, when compared to wild-type KSR2, almost all *KSR2* variants impaired glucose oxidation and palmitate-stimulated fatty acid oxidation (FAO) in transfected cells. These observations indicate that multiple molecular and cellular mechanisms underlie the phenotype associated with disruption of KSR2 in humans, which is characterized by hyperphagia, low basal metabolic rate, obesity, and severe insulin resistance.

RESULTS

Identification of *KSR2* Variants in Obese Individuals

To comprehensively address whether genetic variants in *KSR2* contribute to obesity, we sequenced the coding region and intron/exon boundaries of *KSR2* in 1,770 individuals of mixed European descent with severe, early-onset obesity (mean body mass index [BMI] standard deviation score 3.5; age of onset <10 years) who were recruited to the Genetics of Obesity Study (GOOS) (Farooqi et al., 2003). We also reviewed *KSR2* sequence data obtained from whole-exome sequencing of 331 additional unrelated individuals from the GOOS cohort. We compared the data on individuals with severe obesity to that obtained from sequencing 1,536 control individuals from a large UK population-based study, the ELY study (Williams et al., 1995).

A total of 2.1% of GOOS versus 1.0% of controls had a rare (minor allele frequency (MAF) < 0.5%) frameshift, nonsense or missense variant in *KSR2* (Figure 1A and Table S1 available online). We identified 27 different rare variants in 45/2,101 unrelated severely obese individuals screened compared to 7 different rare variants in 16/1,536 controls (Figure 1A and Table S1; Fisher's exact test using different MAF thresholds adjusted by permutation, p = 0.0025). A combined analysis including a replication sample of 238 cases and 1,117 controls (total 2,339 cases and 2,653 controls), resulted in a permutation adjusted Fisher's exact test p value = 0.0063 (Table S2).

Most variants were found in heterozygous form, although one severely obese individual was homozygous for two *KSR2* variants (R253W and D323E). Twenty-three of the variants identified were found only in severely obese individuals, and many were predicted to be functionally deleterious and altered highly conserved residues (Figure S1), suggesting they may have functional consequences. Five nonsynonymous variants were found in obese cases but also in controls in this study (Figure 1A and Table S1). Three variants were found only in controls, all of whom were overweight/obese (Figure 1A). We compared the frequency of rare variants in severely obese cases and normal weight controls only (n = 1,353). Using a Fisher's exact test and permutation to adjust for using different MAF thresholds for rare variants, we found that there was significant enrichment for rare variants in *KSR2* in severely obese cases (n = 45/2,101) versus normal weight controls (n = 14/1,353); p = 0.0021.

We sequenced *KSR2* in available family members (n = 44) of severely obese probands. Eighteen of the 19 variant carriers were overweight or obese (Table S1), although variants did not consistently cosegregate with obesity in families in a classical Mendelian manner. These findings suggest that *KSR2* variants predispose to obesity against a background of other genetic and environmental factors.

KSR2 Mutations Disrupt Signaling through the Ras-Raf-MEK-ERK Pathway

We next performed a series of studies to explore the molecular mechanisms by which mutations found in severely obese individuals might disrupt KSR2 function. We investigated the impact of the mutations on KSR2's ability to facilitate signaling through the Raf-MEK-ERK pathway. Due to the large number of variants identified (Figure 1B), we focused on variants that disrupt (frameshift/nonsense) or are likely to disrupt (missense mutations in or very close to) the highly conserved kinase domain. There was significant enrichment for variants in this domain in severe obesity (17/2,101) compared to nonobese controls (3/1,353) using Fisher's exact test (p = 0.036).

As human KSR2 has not previously been functionally characterized, we studied two isoforms, KSR2_950 and KSR2_921, which differ in their first 29 amino acids. Both KSR2 isoforms displayed a similar diffuse cytoplasmic distribution, with localization also detected at the plasma membrane (data not shown). KSR2_921 was expressed at a lower level and had a reduced ability to bind B-Raf and facilitate MEK and ERK phosphorylation. All subsequent experiments were therefore performed with KSR2_950.

Using confocal microscopy, we investigated the intracellular localization of WT and mutant KSR2 expressed in Cos7 cells and its colocalization with endogenous B-Raf, MEK, or ERK. None of the mutations affected the intracellular localization of KSR2 (Figure S2). WT KSR2 colocalized with B-Raf, MEK, and ERK in the cytoplasm and at the plasma membrane and this was unaffected by the mutations (Figures 2A and S2).

To determine whether KSR2 mutations affect Ras-Raf-MEK-ERK signaling, HEK293 cells were transfected with Flag-tagged WT and mutant forms of KSR2 and stimulated with EGF. Using

(B) Schematic representation of full-length KSR2 (Q6VAB6), indicating the location of each of the mutations identified in individuals with severe early-onset obesity and those found in controls (shown in gray). Domains conserved in KSR1 are indicated in blue (CA1-4) and the domain found only in KSR2 is shown in green; the kinase domain is indicated in red. The mutations found in severely obese individuals that were subjected to functional characterization are shown in red. See also Tables S1 and S2 and Figure S1.

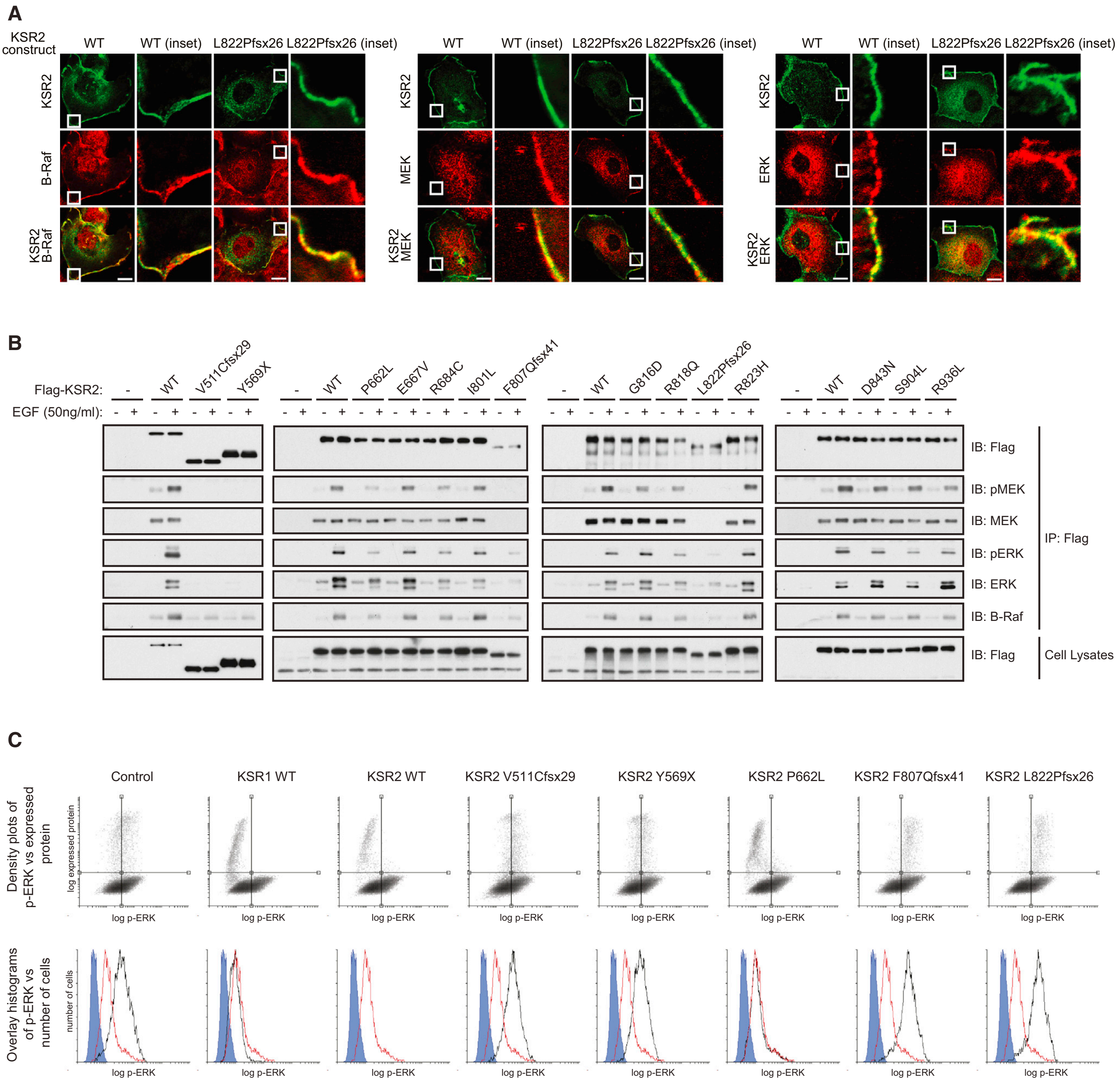

Figure 2. Obesity-Associated *KSR2* Mutations Disrupt Raf/MEK/ERK Signaling

(A) Confocal microscopy of EGF-stimulated Cos7 cells showing the colocalization of transiently expressed KSR2 WT and the frameshift mutation, L822Pfsx26 (green), with endogenous B-Raf, MEK, and ERK (red) in the cytoplasm and plasma membrane (inset). Scale bars, 10 μm.

(B) HEK293 cells transfected with the indicated KSR2 constructs were serum starved for 16 hrs prior to stimulation with 50 ng/ml EGF for 10 min. Lysates were subjected to immunoprecipitation with Flag-agarose and immunoblotted with the indicated antibodies.

(C) Top: Flow cytometry density plots showing the effect of KSR overexpression on ERK phosphorylation. Bottom: Flow cytometry histograms gated on transfected cells, comparing the effect on ERK phosphorylation of overexpressing different KSR2 mutants (black) versus WT KSR2 (Red). A background control is shown in blue. See also Figure S2.

coimmunoprecipitation, we found that WT KSR2 was constitutively bound to MEK, consistent with previous reports (Denouel-Galy et al., 1998; Dougherty et al., 2009; Müller et al., 2000; Yu et al., 1998) and, upon EGF stimulation, interacted with ERK and B-Raf (Figure 2B). In addition, phosphorylation of the KSR2-associated MEK and ERK could be detected. In contrast, two mutations that result in truncated versions of KSR2 that lack the kinase domain, V511Cfsx29 and Y569X, were unable to interact with MEK and ERK and displayed a significantly reduced ability to bind B-Raf. A similar result was

also seen with two additional frameshift mutants, suggesting that all four mutations result in a nonfunctional form of KSR2 (Figure 2B). The remaining missense mutations in or near the kinase domain expressed at a similar level to WT KSR2 and retained their ability to interact with MEK. However, the phosphorylation of associated MEK was reduced with the P662L, R684C, G816D, and R818Q mutants, while that of ERK was reduced with the P662L, R684C, and R818Q mutants. In addition the P662L, R684C, and R818Q mutants reduced the ability of KSR2 to interact with B-Raf (Figure 2B).

While studying the colocalization of KSR2 with components of the MAPK signaling pathway, we noticed that overexpression of KSR2 in Cos7 cells led to reduced levels of phosphorylated ERK. This observation is consistent with previous studies on KSR1, which showed that overexpression of KSR1 results in attenuated ERK activation (Joneson et al., 1998). We used flow cytometry to measure the effect of overexpressing wild-type and mutant KSR2 on ERK phosphorylation. Cells transfected with WT KSR2 were found to have a substantial reduction in ERK phosphorylation compared to a control protein. In contrast, the V511Cfsx29, Y569X, F807Qfsx41, and L822Pfsx26 mutants did not reduce ERK activation, providing further evidence that they are nonfunctional (Figure 2C).

Mutations in KSR2 Disrupt Interactions between the Kinase Domain, B-Raf, and MEK

We modeled the mutations using the structure of the KSR2 kinase domain in complex with MEK (Brennan et al., 2011) (Figures 3A and 3B). Of the three frameshift mutants, V511Cfsx29 results in a truncated protein that not only lacks the ERK-binding domain but also the entire kinase domain leading to a completely nonfunctional KSR2 molecule (Figure S3). Similarly, the Y569X nonsense mutation also removes the kinase domain. F807fsx41 and L822Pfsx26 fall within the activation segment of the KSR2 kinase domain, resulting in the loss of the APE motif, a conserved feature of all protein kinases that is involved in substrate binding, and also the remainder of the kinase domain, including the αG helix (Figure S3). This would be expected to completely destabilize the interaction of KSR2 and MEK and explains our finding that none of the frameshift mutants, nor the nonsense mutant, are able to interact with MEK (Figure 2B).

The P662L, E667V, and R684C mutations lie within the N-lobe of the kinase domain, in a region involved in binding B-Raf (Figure 3A). P662 is directly involved in the interaction with B-Raf (Figure 3C), and R684 forms a long electrostatic contact to Glu664 of B-Raf (Figure 3D), explaining how mutation of these residues leads to reduced B-Raf binding. The remaining point mutations are located in the C-lobe of the catalytic domain. I801 lies close to the DFG motif within the activation segment (Figure 3E); G816 and R818 also lie in the activation segment, although this region (808–819) is disordered in the KSR2 kinase domain structure. Additionally, R823 and D843 lie close to the KSR2-MEK interface (Figures 3F and 3G), and thus mutations of these residues may disrupt KSR2-MEK interactions. S904 is not directly involved in binding to MEK and B-Raf (Figure 3H); however, a Leu substitution at this position would cause a steric clash with Leu894 and likely destabilize the C-lobe of the KSR2 kinase domain. R936L is C-terminal to the ordered region of the kinase domain and so was not studied. Cumulatively, these findings indicate that the nonsense, frameshift, and some of the point mutations disrupt key interactions between KSR2 and B-Raf and MEK.

Human Phenotype Associated with Loss-of-Function *KSR2* Mutations

We next sought to determine the phenotype associated with loss-of-function mutations in *KSR2* in humans. Eighteen probands and family members harboring *KSR2* variants (mean BMI ± SEM = 35.4 ± 7.5 kg/m^2) consented to take part in clinical studies (Table S1). We compared their data to 26 equally obese volunteers recruited from the local community (mean BMI 36.4 ± 7.0 kg/m^2) in whom variants in *KSR2* were excluded by sequencing.

Final height of male (176.1 ± 3.2 cm) and female (165.8 ± 1.2 cm) *KSR2* mutation carriers was not significantly different from obese controls (176.3 ± 1.9 cm and 165.2 ± 1.8 cm, respectively), suggesting that heterozygous loss-of-function *KSR2* mutations do not adversely affect linear growth. We measured body composition by dual energy X-ray absorptiometry (DEXA). The mean percentage body fat of *KSR2* mutation carriers (47.0% ± 2.0%) was similar to that of obese controls (45.8% ± 1.2%). There was a history of increased food-seeking behavior in childhood and energy intake at an 18MJ ad libitum test meal given to children after an overnight fast was significantly greater than that of 14 normal weight children (Figure 4A). However, hyperphagia was reported as being less prominent with age and in adult *KSR2* mutation carriers, measured ad libitum energy intake did not differ significantly from obese controls (Figure 4B).

Basal metabolic rate (BMR) measured by indirect calorimetry correlates very closely with energy expenditure predicted on the basis of age, gender, and body composition (Cunningham, 1991) in normal weight and obese healthy subjects and in individuals with genetic forms of obesity affecting leptin-melanocortin signaling (Farooqi et al., 2007; Greenfield et al., 2009). We measured BMR in *KSR2* mutation carriers in thermoneutral conditions after a 10 hr supervised fast. Measured BMR was less than predicted BMR in children with *KSR2* mutations (8.0 ± 0.7 MJ/day versus 8.3 ± 0.7 MJ/day), although this was not statistically significant. However, measured BMR was significantly reduced compared to predicted BMR in adult mutation carriers (Figure 4C). In addition, the respiratory quotient (RQ; ratio of carbohydrate to fat oxidation) was increased in *KSR2* mutation carriers compared with obese controls (Figure 4D).

We measured heart rate and heart rate variability using a portable digital accelerometer, a noninvasive device for quantifying autonomic nervous system activation. Heart rate was measured continuously for 24 hr in adults only, as heart rate cannot readily be compared across children of different ages. Sleeping heart rate was significantly lower in *KSR2* mutation carriers compared to obese controls (Figure 4E; $p = 0.01$). The increase in heart rate with waking was comparable in both groups (Figure 4E). There were no differences in the root mean square of successive differences (RMSSD), low-frequency (LF) power or high-frequency (HF) power components between the two groups (data not shown), suggesting that autonomic tone is preserved in *KSR2* mutation carriers. Twenty-four hour urinary

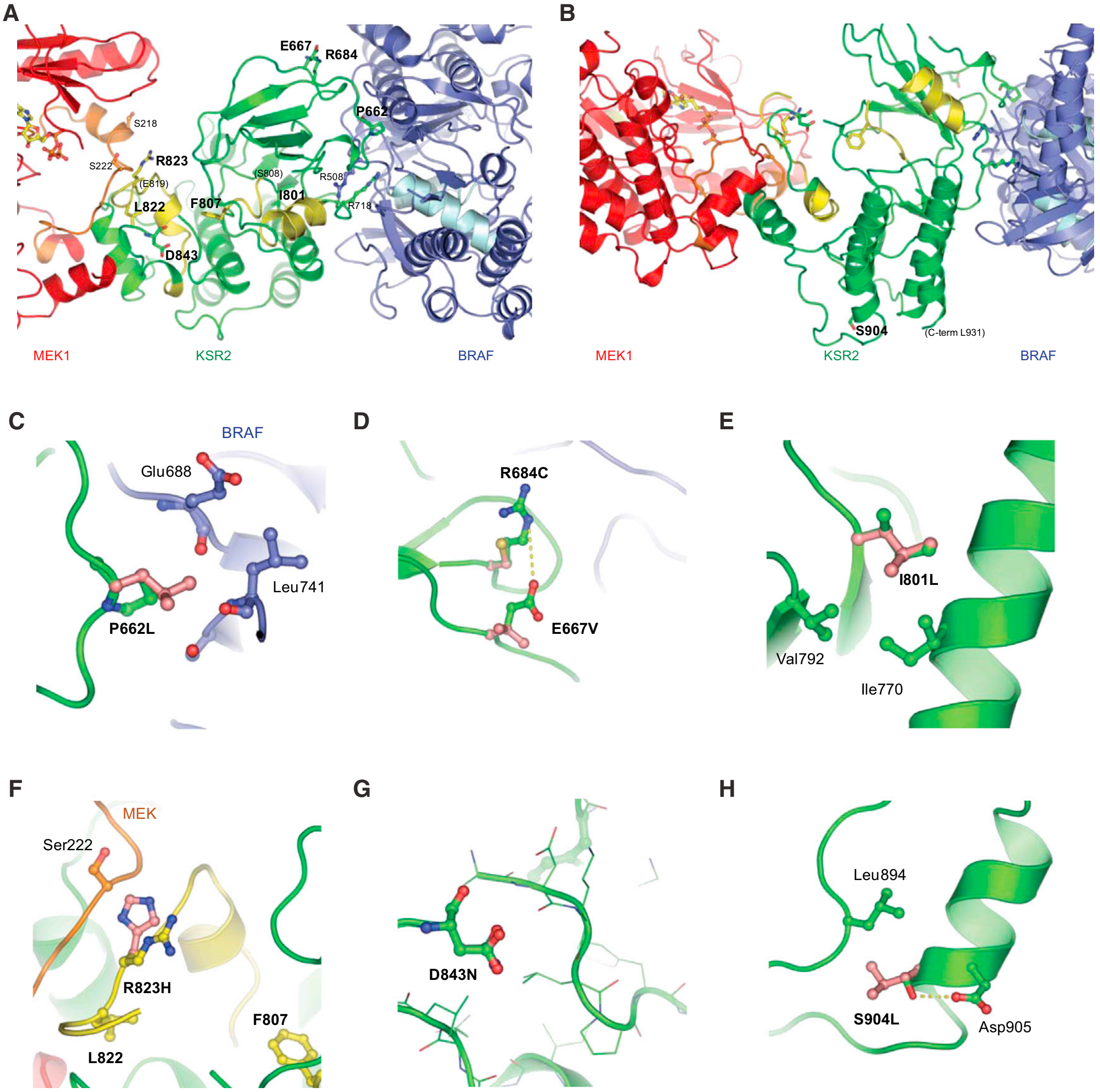

Figure 3. Structural Modeling of Mutations within the Kinase Domain of KSR2

(A and B) Structure of the MEK1-KSR2-BRAF ternary complex (Brennan et al., 2011) indicating the location of all the mutations studied.

(C–H) Local environment and packing interactions of specific amino acid substitutions in KSR2. Disease-associated residues are shown in pink. See also Figure S3.

norepinephrine, epinephrine, and dopamine excretion, thyroid-stimulating hormone, and free thyroxine concentrations were in the normal range (Table S3). There was no difference in temperature between *KSR2* mutation carriers and obese controls (36.5 ± 0.09°C versus 36.4 ± 0.09°C, respectively).

Two *KSR2* mutation carriers were excluded a priori from studies due to a diagnosis of type 2 diabetes. Fasting plasma insulin levels were significantly increased in *KSR2* mutation carriers compared to obese controls (Figure 4F) as were C-peptide levels (1,922 ± 120 versus 1,015 ± 77 pmol/l; $p < 0.0001$), consistent with increased insulin secretion. In addition, *KSR2* mutation carriers exhibited an increased insulin response to a 75 g oral glucose load (Figure 4F). While there was no significant difference in fasting glucose levels between the groups, *KSR2* mutation carriers exhibited impaired glucose tolerance (Figure 4G). All mutation carriers had clinical evidence of acanthosis nigricans; hyperpigmentation of axillary and flexural skin, which is a recognized marker of severe insulin resistance. Mean systolic and diastolic blood pressures, fasting cholesterol, triglycerides, and adiponectin levels were comparable to those seen in obese controls (Table S3).

Increased Energy Intake and Decreased Energy Expenditure in *Ksr2* Null Mice

To further investigate how energy homeostasis is perturbed when KSR2 is disrupted, we performed additional studies in $Ksr2^{-/-}$ mice. Male and female $Ksr2^{-/-}$ mice were hyperphagic and rapidly gained body weight, in particular fat mass, when fed ad libitum (Figures 5A–5D; Table S4). To assess the relative

contribution of hyperphagia to the weight gain of *Ksr2*$^{-/-}$ mice, we limited their food intake to the amount consumed by wild-type mice (pair feeding [PF]). We found that despite being fed the same amount of diet as *Ksr2*$^{+/+}$ littermates from weaning, *Ksr2*$^{-/-}$ mice gained more weight. This excess weight was predominantly due to an increase in fat mass (Figures 5C and 5D; Table S4). These data demonstrate that, in keeping with our observations in humans with *KSR2* mutations, both increased energy intake and reduced energy expenditure contribute to the obesity of *Ksr2*$^{-/-}$ mice.

We sought to determine whether the energy expenditure defect seen in *Ksr2*$^{-/-}$ mice could be explained by impaired mitochondrial FAO. As the inability to maintain body temperature when placed in a cold environment is a sensitive indicator of impaired FAO in rodents (Guerra et al., 1998; Schuler et al., 2005), we challenged additional cohorts of *Ksr2*$^{-/-}$ mice by housing them at 4°C for up to 4 hr. Ad libitum fed male and female *Ksr2*$^{-/-}$ mice were cold tolerant at 6 weeks of age (Figures 5E, 5F, and S4) but became significantly cold intolerant by 10 weeks of age (Figures 5G, 5H, and S4).

Human *KSR2* Mutations Impair Glucose Oxidation and Fatty Acid Oxidation in Cells

Studies using mouse embryonic fibroblasts (MEFs) from *Ksr2*$^{-/-}$ mice have suggested that the interaction between KSR2 and AMPK may contribute to their abnormal energy homeostasis and metabolism (Costanzo-Garvey et al., 2009). To investigate the potential interaction between AMPK and human KSR2, we performed immunoprecipitation of endogenously expressed AMPK from HEK293 cells overexpressing Flag-tagged WT and mutant KSR2. While all human KSR2 mutants bind AMPK, the frameshift mutations and nonsense mutation that disrupt the kinase domain and the missense mutant E667V, bound AMPK with reduced affinity (Figure 6A). As the CA3 region and amino acids located between the CA2 and CA3 regions contribute to the ability of KSR2 to interact with the AMPKα subunit, we studied three additional mutations that lie within/close to this domain (D323E, A373T, and R397H). One of these, A373T, exhibited reduced AMPK binding (Figure 6A).

Activation of AMPK stimulates insulin-independent glucose oxidation and inhibits the synthesis of long-chain fatty acids by inhibiting acetyl-CoA carboxylase (ACC), thereby promoting the beta-oxidation of long-chain fatty acids by enabling the import of fatty acyl-CoA molecules into mitochondria. In previous studies, ectopic expression of Ksr2 in *Ksr2*$^{-/-}$ MEFs increased basal glucose uptake and palmitate-stimulated oxygen consumption rate, a marker of FAO (Costanzo-Garvey et al., 2009). We investigated the impact of human *KSR2* mutations on glucose oxidation using C2C12 cells overexpressing WT and mutant forms of KSR2. Measurements of oxygen consumption rate (OCR), as an indicator of oxidative phosphorylation, and extracellular acidification rate (ECAR) rate, as an index of glycolysis (i.e., lactate production), were performed (Figure S5). We found that all mutations significantly reduced glucose oxidation compared to WT KSR2 (Figure 6B).

To evaluate the role of *KSR2* mutations in FAO, C2C12 cells overexpressing KSR2 mutants were differentiated into myocytes and OCR was measured prior to the addition of either 100 μM palmitate complexed with BSA, or BSA alone (Figures 6C and S5). We found that while cotransfection of WT KSR2 increased palmitate-stimulated oxygen consumption, this effect was reduced by all KSR2 mutants with the exception of I801L (Figure 6C). There is no obvious structural/molecular explanation for why I801L affects glucose, but not fatty acid, oxidation. Together, these data demonstrate that, when studied in cells, *KSR2* mutations are associated with disrupted fuel utilization.

Clinical reports suggested that some *KSR2* mutation carriers experienced marked weight loss in childhood when prescribed the antidiabetic drug metformin (for severe insulin resistance). Metformin (1,1-dimethylbiguanide) is the most widely prescribed drug used in type 2 diabetes and can stimulate AMPK activation (Hardie et al., 2012). We investigated the change in FAO seen with the KSR2 mutants that previously had the greatest impact upon FAO in response to 1 mM metformin (Figure 6D). We found that preincubation with metformin increased palmitate-stimulated oxygen consumption in untransfected cells. Transfection of WT KSR2 increased the basal level of FAO, which increased further with metformin. The reduced basal level of FAO seen with the KSR2 mutations was completely rescued in all cases by the addition of metformin.

DISCUSSION

In this study, we linked the development of early-onset obesity and severe insulin resistance in humans with rare variants in *KSR2*, which affect its functional properties. When expressed in cells, almost all of the KSR2 mutations impaired FAO and glucose oxidation. In addition, several mutants disrupted Raf-MEK-ERK signaling.

Most of the rare variants identified in severely obese individuals affected highly conserved residues, were not found in normal weight controls sequenced using the same methods, and were associated with loss of function in a number of assays. Therefore, we conclude that these *KSR2* variants are likely to be major drivers of the phenotype seen in the affected individuals. Although rare loss-of-function variants in *KSR2* are highly enriched in cases versus controls, they do not consistently cosegregate with severe obesity and as such are likely to interact with other genetic and/or environmental factors to modulate the phenotype within and between families. Additional genetic studies will be needed to determine whether these findings can be replicated in comparable cohorts.

Some of the variants found in severely obese individuals were also found in controls in this study; although notably some of these controls were also overweight or obese. In addition, some *KSR2* variants have recently been reported in publicly available exome data (Table S1). However, as BMI and additional phenotypic information for individuals in these data sets are not available, the precise contribution of these variants to obesity remains to be established.

KSR2 Regulates Energy Intake and Expenditure in Mouse and Man

Our physiological data demonstrate that KSR2 is a regulator of food intake, basal metabolic rate, FAO, and glucose oxidation in humans. While to date, other highly penetrant genetic forms

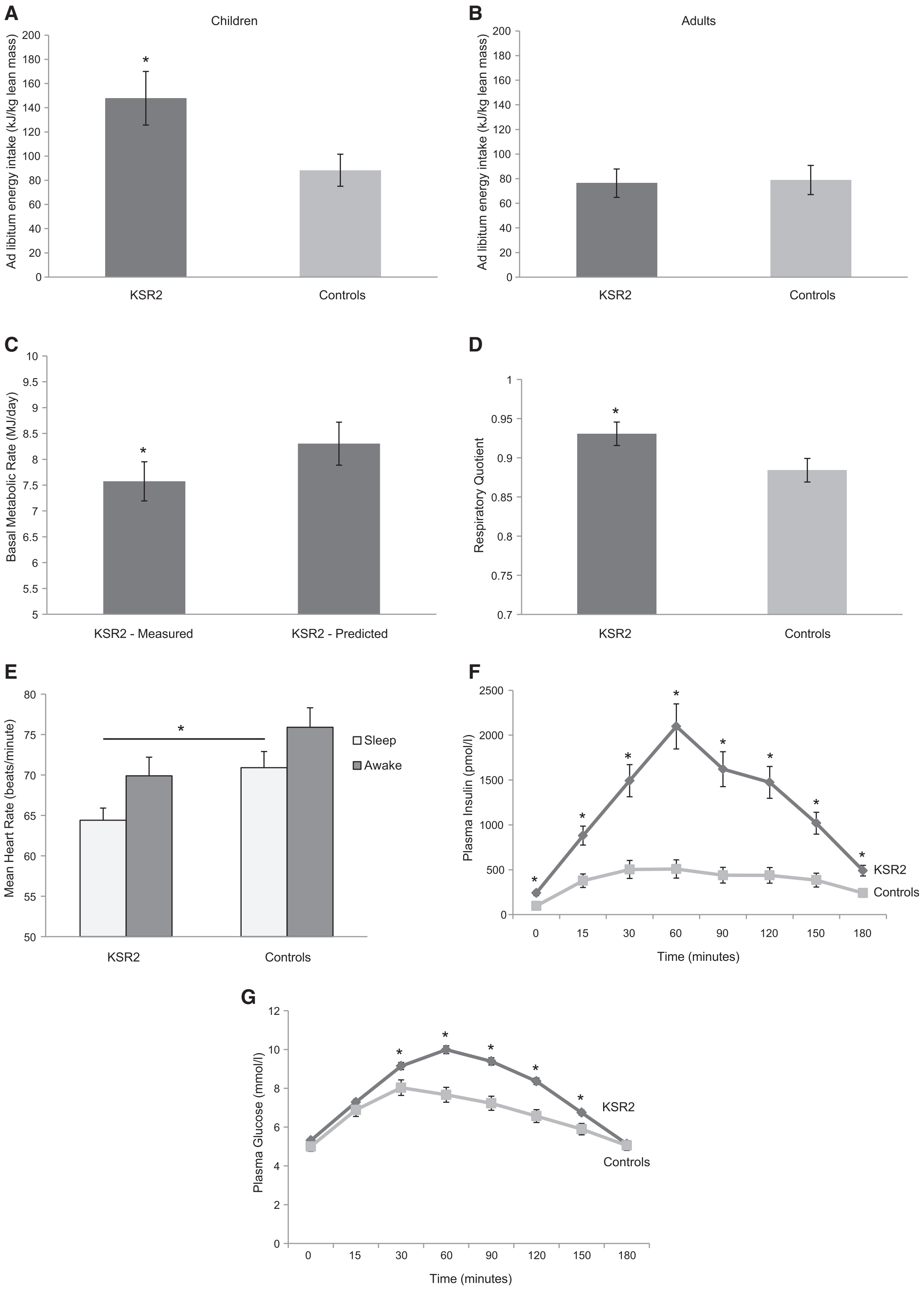

Figure 4. *KSR2* Mutations Affect Energy Intake and Energy Expenditure in Humans

Data are presented for individuals carrying rare variants in *KSR2* and obese controls in whom *KSR2* variants were excluded. Values are mean ± SEM; *p < 0.05.

(A and B) Ad libitum energy intake at an 18MJ test meal presented after an overnight fast to children with *KSR2* mutations and normal weight children (A) and to adults with *KSR2* mutations compared to obese controls (B); intake is expressed as kilojoules per kilogram of lean mass.

(legend continued on next page)

of obesity have predominantly been associated with hyperphagia (Farooqi and O'Rahilly, 2006), *KSR2* mutations are in addition associated with low BMR and reduced heart rate when tested under the same experimental conditions as individuals with other genetic forms of obesity (Farooqi et al., 2007; Greenfield et al., 2009).

BMR accounts for approximately 70% of energy expenditure in humans, with the remainder being attributable to voluntary physical activity (20%) and nonexercise related activity thermogenesis (10%). Impaired sympathetic nervous system (SNS) activation is a possible explanation for the low BMR observed, but we consider this is unlikely given that RMSSD, LF power, and HF power measurements of heart rate data were comparable to measurements in obese controls. Furthermore, urinary norepinephrine excretion was in the normal range in mutation carriers, in contrast to measurements in individuals with other genetic forms of obesity characterized by impaired SNS activation (Greenfield et al., 2009). However, these measurements have their limitations, and further studies will be needed to measure sympathetic innervation and activation of adipose tissue, in particular brown adipose tissue, which plays a key role in thermogenesis and energy expenditure (Lowell and Spiegelman, 2000). Studies in additional *KSR2* mutation carriers will be needed to replicate these findings, establish whether loss-of-function mutations impact on other components of energy expenditure and to investigate whether a genotype/phenotype correlation exists.

There is considerable overlap between the phenotypes we observed in humans and those seen in *Ksr2*$^{-/-}$ mice. *Ksr2*$^{-/-}$ mice become markedly obese after weaning while heterozygous mice developed more modest, later-onset obesity (Revelli et al., 2011). Our findings support a major role for hyperphagia as well as reduced metabolic rate in the development of obesity associated with Ksr2 deficiency. Although Ksr2 is expressed in areas of the hypothalamus known to be involved in mediating leptin's effects on food intake, such as the arcuate nucleus (Figure S4), previous studies performed in *Ksr2*$^{-/-}$ mice did not convincingly link leptin and melanocortin pathways to the obesity phenotype (Revelli et al., 2011). We found that *Ksr2*$^{-/-}$ mice tolerated cold exposure at 6 weeks of age, when we saw little evidence for a defect in energy expenditure; however, they were modestly cold intolerant at 10 weeks of age, when weight gain occurred despite pair-feeding. Previous studies have suggested that the decreased energy expenditure seen in *Ksr2*$^{-/-}$ mice is not explained by decreased physical activity (Costanzo-Garvey et al., 2009; data not shown).

KSR2 Mutations Disrupt Multiple Signaling Pathways

While some of the rare *KSR2* variants identified in this study may affect the function of the protein through mechanisms that were not tested here, we show that rare obesity-associated *KSR2* mutations disrupt multiple molecular processes when studied in cells. We found that all the frameshift mutants, the nonsense mutant and several missense mutations reduced the ability of KSR2 to facilitate signaling within the Raf-MEK-ERK pathway. The assays used in these studies identified variants that were clearly deficient in activity but may not have been able to differentiate variants with mild loss of function.

Modulation of the intensity and duration of ERK signaling may be a mechanism by which *KSR2* mutations are associated with metabolic disease. Diet-induced obesity in mice and humans is associated with increased ERK1 activity in adipose tissue, whereas *Erk1*$^{-/-}$ mice appear resistant to diet-induced obesity and remain insulin sensitive on a high-fat diet (Bost et al., 2005). Leptin-deficient *ob/ob* mice crossed with *Erk1*$^{-/-}$ mice are partially protected against hyperglycemia and hepatic steatosis despite developing severe obesity (Jager et al., 2011), suggesting that ERK signaling modulates insulin sensitivity independent of its potential direct effects on adipose tissue.

Ex vivo experiments suggest a role for KSR2 in liver, adipose tissue, and skeletal muscle, tissues in which KSR2 mRNA has been detected at low levels (Costanzo-Garvey et al., 2009). However, KSR2 protein is detectable by western blot only in the brain (Figure S4). It therefore seems likely that most of the physiological effects of *KSR2* mutations may be mediated by central nervous system pathways. Ras-Raf-MEK-ERK signaling is required for normal brain development (Lanctot et al., 2013) and a growing body of evidence suggests that neurotrophin-mediated signaling through this pathway is involved in synaptic plasticity (Canal et al., 2011; Shalin et al., 2006). Deletion and reactivation of components of KSR2-mediated signaling in targeted neuronal populations will be needed to dissect the full functional repertoire of centrally expressed KSR2.

We have shown that mutations associated with human obesity and insulin resistance impair KSR2's ability to stimulate fatty acid and glucose oxidation, in transfected cells. The interaction of human KSR2 with AMPK suggests a plausible mechanism that may contribute to some of the effects we observed in cells and in vivo, although the precise pathways involved are likely to be highly complex, tissue-specific, and altered by environmental factors such as diet and exercise. In rodents, AMPK activation in hypothalamic neurons can be modulated by peripheral nutritional signals such as the anorectic hormone leptin (Minokoshi et al., 2004; Yang et al., 2011) and the orexigenic hormone ghrelin (Kola et al., 2005). Direct injection of pharmacological activators of AMPK into the hypothalamus promotes feeding (Andersson et al., 2004), whereas disruption of AMPKα2 in neurons expressing anorectic POMC-derived peptides leads to obesity due to reduced energy expenditure and increased food intake (Claret et al., 2007).

Further studies will be needed to investigate the contribution of downstream components of the KSR2-AMPK signaling complex to energy homeostasis and glucose control. AMPK regulates mTORC1 (mammalian target of rapamycin complex 1) via direct phosphorylation of the tumor suppressor TSC2 and the mTORC1 subunit Raptor, leading to inhibition of mTORC1

(C) Measured and predicted basal metabolic rate (BMR) adjusted for kg fat free mass in adults with *KSR2* mutations.

(D) Respiratory quotient as measured by indirect calorimetry.

(E) Heart rate (beats per minute) during sleep and in the awake state measured using a portable digital accelerometer.

(F and G) Plasma insulin (pmol/l) and glucose (mmol/l) before and after a 75 g oral glucose load (given at time 0). See also Table S3.

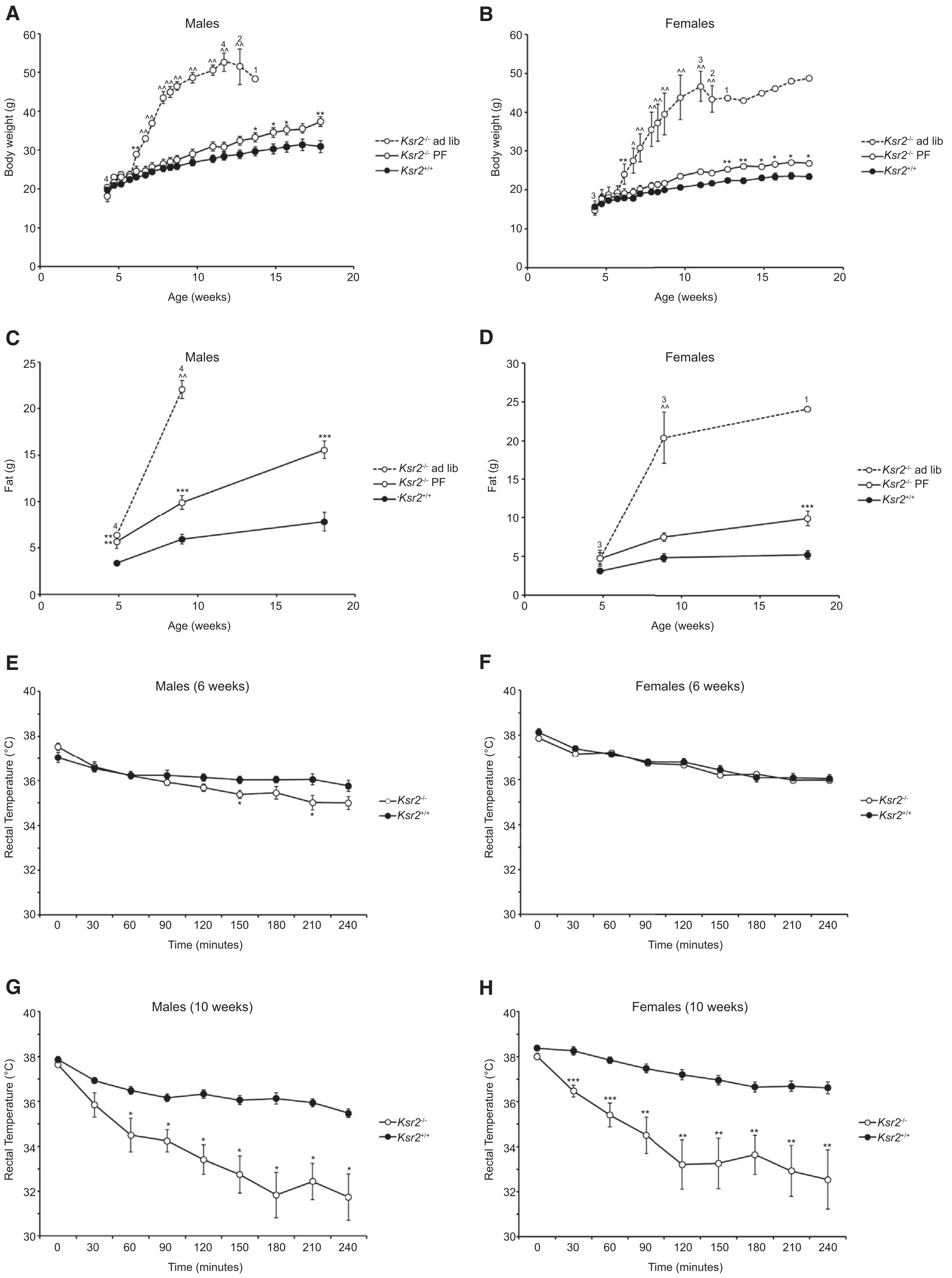

Figure 5. The Obesity of *Ksr2*$^{-/-}$ Mice Is Due to Increased Energy Intake and Decreased Energy Expenditure and Is Associated with Cold Intolerance

Body weight of *Ksr2*$^{-/-}$ mice and WT littermates in response to ad libitum feeding and a pair-feeding paradigm.

(A) At weaning, male mice (12 *Ksr2*$^{+/+}$ and 4 *Ksr2*$^{-/-}$) were fed ad libitum. 9 *Ksr2*$^{-/-}$mice were pair-fed (PF) the amount of diet consumed ad libitum by *Ksr2*$^{+/+}$ mice. Although only 1 of 7 *Ksr2*$^{-/-}$ mice fed ad libitum survived to 15 weeks of age, all pair-fed mice survived to 18 weeks of age. Numbers above the *Ksr2*$^{-/-}$ ad libitum group data reflect the number of *Ksr2*$^{-/-}$ ad libitum mice alive at the time of the body weight measurement; all other mice survived to the end of the study.

(legend continued on next page)

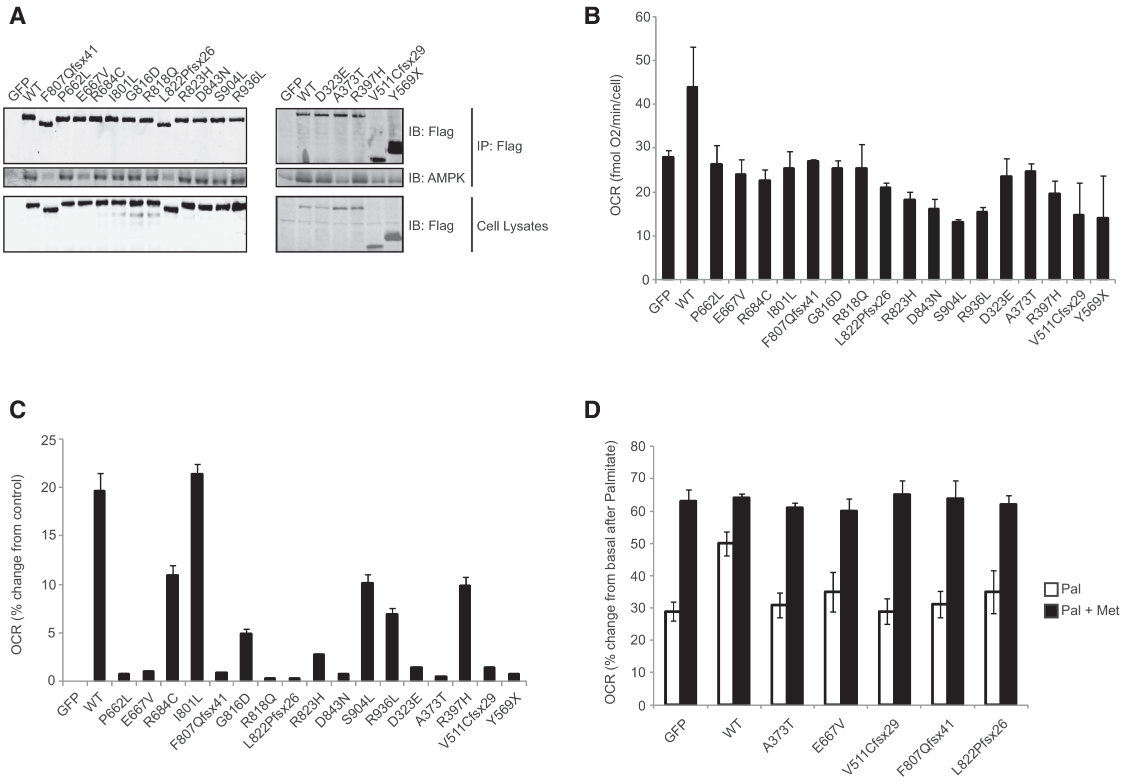

Figure 6. Human *KSR2* Mutations Are Associated with Reduced Glucose and Fatty Acid Oxidation in Transfected Cells

(A) Lysates from HEK293 cells transfected with the indicated KSR2 constructs were subjected to immunoprecipitation with Flag-agarose and immunoblotted with the indicated antibodies.

(B) Measurement of glucose oxidation in C2C12 cells using the Seahorse XF24 extracellular flux analyzer. C2C12 cells overexpressing wild-type and mutant forms of KSR2 were stimulated with 2 μM FCCP and oxygen consumption rate (OCR) measured. Data were normalized to cell count by the sulphorhodamine B (SRB) assay.

(C) Measurement of FAO in differentiated C2C12 cells using the Seahorse XF24 extracellular flux analyzer. C2C12 cells overexpressing wild-type and mutant forms of KSR2 were stimulated with 100 μM palmitate and OCR measured. Values represent the means of three independent experiments. Error bars represent SEM. The data are represented as % change from GFP-transfected control cells.

(D) Effect of metformin on FAO. C2C12 myocytes overexpressing wild-type and mutant forms of KSR2 were stimulated with 100 μM palmitate (Pal) and OCR measured. Cells were preincubated with 1 mM metformin (Met) for 1 hr prior to the start of the assay and metformin was present in the reaction buffer during the assay. The data are represented as percentage (%) change from GFP transfected control cells. See also Figure S5.

activity (Shaw, 2009). As hyperactivation of mTORC1 in POMC neurons leads to hyperphagia and obesity in mice (Mori et al., 2009), the increased phosphorylation of mTORC1 seen in the hypothalamus of *Ksr2*$^{-/-}$ animals (Revelli et al., 2011), may contribute to their increased food intake. Depletion of KSR2 by shRNA in MIN6 cells that express endogenous KSR2, is associated with a reduction in phosphorylated AMPK, ACC and Raptor (Fernandez et al., 2012). However, these effects may be cell-line dependent as we found that WT and mutant KSR2 had no effect on AMPK activation and TSC2 and Raptor phosphorylation in HEK293, Cos7, or C2C12 cells in which endogenous KSR2 cannot be detected (Figure S5 and data not shown).

The anecdotal clinical observation that metformin treatment leads to weight loss in some *KSR2* mutation carriers needs to be investigated further in a prospective, controlled clinical experimental study with measurement of BMR, RQ, and insulin sensitivity. Metformin improves insulin sensitivity, lowers blood glucose and cholesterol, and reduces body weight in some

(B) At weaning, female mice (10 *Ksr2*$^{+/+}$ and 3 *Ksr2*$^{-/-}$) were fed ad libitum. 6 *Ksr2*$^{-/-}$ mice were pair-fed (PF) to the amount of chow consumed ad libitum by *Ksr2*$^{+/+}$ mice.

(C and D) Fat mass of pair-fed male and female mice.

(E) Rectal temperatures of 6 week-old male *Ksr2*$^{+/+}$ (n = 9) and *Ksr2*$^{-/-}$ (n = 10) mice following cold exposure at 4°C.

(F) Rectal temperatures of 6 week-old female *Ksr2*$^{+/+}$ (n = 10) and *Ksr2*$^{-/-}$ (n = 9) mice following cold exposure at 4°C.

(G) The same male mice presented in (E) were studied again at 10 weeks of age.

(H) The same female mice presented in (F) were studied again at 10 weeks of age. Differences from WT: *$p < 0.05$, **$p < 0.01$, ***$p < 0.001$; KO-ad lib different from KO-PF and WT: ∧ $p < 0.01$, ∧∧ $p < 0.001$. Error bars represent SEM.

See also Table S4 and Figure S4.

individuals with type 2 diabetes, although the underlying mechanisms of action, which include modulating AMPK activation, remain poorly understood (Viollet et al., 2012). We assessed the ability of WT and mutant forms of KSR2 to affect AMPK phosphorylation in the presence or absence of 1 mM metformin and observed that metformin leads to phosphorylation of AMPK Thr172 equally in cells transfected with WT and mutant forms of KSR2 (Figure S5).

Further studies will be necessary to understand the extent to which AMPK activation contributes to the full repertoire of metformin's effects and its ability to ameliorate the effects of *KSR2* mutations on substrate utilization. These observations and our in vitro findings suggest that pharmacological approaches based on the modulation of KSR2 activity could represent a novel potential therapeutic strategy for the treatment of obesity and type 2 diabetes.

EXPERIMENTAL PROCEDURES

Subjects and Ethical Permissions

DNA samples were obtained from individuals with severe early-onset obesity from the GOOS cohort or from control individuals from the ELY study. Each subject (or their parent for those under 16 years) provided written informed consent; minors provided oral consent. Details of the phenotyping studies are described in the Extended Experimental Procedures. Normal weight children (n = 14) and obese adults (n = 26) were recruited for these studies by advertisement in the local media. All participants were weight-stable nonsmokers and were not taking medications that could affect energy homeostasis, gut absorption, or lipid/carbohydrate metabolism.

Immunoprecipitation and Immunoblotting

Cell lysates were immunoprecipitated using anti-Flag M2 affinity gel (Sigma) and samples analyzed using SDS-PAGE and western blotting.

Immunofluorescence and Flow Cytometry

Fixed Cos7 cells on glass coverslips were immunostained and imaged using a Zeiss LSM510 confocal microscope. Fixed HEK293 cells were immunostained for flow cytometry before subsequent analysis on a BD FACS calibur flow cytometer.

Structural Modeling

Modeling of *KSR2* mutations onto the MEK1-KSR2 structure (PDB: 2Y4I) (Brennan et al., 2011) was performed using COOT (Emsley and Cowtan, 2004).

Measurement of Glucose Oxidation and Fatty Acid Oxidation

Measurements were carried out on differentiated C2C12 myotubes using the Seahorse XF^{e}24 analyzer.

Mouse Husbandry

Ksr2 KO mice were maintained as described previously (Revelli et al., 2011). Details of the mouse studies can be found in the Extended Experimental Procedures.

Statistics

Genetic Studies

Fisher's exact test was used to compare the number of rare (MAF < 0.5%) variants between cases and controls. Permutation was used to calculate the p value correcting for multiple testing over different MAF thresholds.

All Other Studies

Data are presented as mean ± SEM throughout. Comparisons between two groups were analyzed by unpaired Student's t test, and comparisons among three groups were analyzed by one-way ANOVA with post hoc analysis performed by the Bonferroni method, using PRISM 4.03 (GraphPad) software. Values were considered statistically significant when $p < 0.05$.

SUPPLEMENTAL INFORMATION

Supplemental Information includes Extended Experimental Procedures, five figures, and four tables and can be found with this article online at http://dx.doi.org/10.1016/j.cell.2013.09.058.

AUTHOR CONTRIBUTIONS

B.B., A.H., E.G.B., I.B., and I.S.F. designed, performed, and analyzed genetic studies. B.B., S.G., E.G., and L.R.P. performed genotyping and Sanger sequencing. L.R.P, N.A., M.C.B., and S.G. designed, performed, and analyzed functional studies. D.B. performed and analyzed structural modeling studies. M.P.M. advised on the design and interpretation of Seahorse analyses. A.A.v.d.K., J.M.K, E.H., and I.S.F. designed, performed, and analyzed clinical studies. J.M.K., E.H., R.B., S.A., P.C.H., J.P.H.S., E.C., S.O.R., and I.S.F. established, managed, and recruited subjects in the GOOS cohort. N.J.W. established, managed and recruited subjects in the ELY cohort. J.-P.R., D.D., S.J.-J., and D.R.P. designed, performed, and analyzed mouse studies. L.R.P., N.A., M.C.B., and I.S.F. wrote the manuscript with contributions from all authors. N.A. and M.C.B. contributed equally to the work.

ACKNOWLEDGMENTS

We are indebted to the participants and their families for their participation and to the physicians involved in the Genetics of Obesity Study (GOOS). We thank Brian Lam for help with flow cytometry; Matthew Gratian, Margaret Robinson, and Patrycia Kozik for microscopy advice and Pamela Pun for assistance using the Seahorse XF analyzer. This work was supported by the Wellcome Trust (098497/Z/12/Z; 077016/Z/05/Z; 096106/Z/11/Z) (ISF and LRP), Medical Research Council (MC_U106179471) (NW), NIHR Cambridge Biomedical Research Centre (ISF, IB and SOR), and European Research Council (ISF). This study makes use of data generated by the UK10K Consortium (WT091310). A full list of the investigators who contributed to the generation of the data is available from http://www.UK10K.org.

Received: July 18, 2013
Revised: July 31, 2013
Accepted: September 20, 2013
Published: October 24, 2013

REFERENCES

Andersson, U., Filipsson, K., Abbott, C.R., Woods, A., Smith, K., Bloom, S.R., Carling, D., and Small, C.J. (2004). AMP-activated protein kinase plays a role in the control of food intake. J. Biol. Chem. *279*, 12005–12008.

Bost, F., Aouadi, M., Caron, L., Even, P., Belmonte, N., Prot, M., Dani, C., Hofman, P., Pagès, G., Pouysségur, J., et al. (2005). The extracellular signal-regulated kinase isoform ERK1 is specifically required for in vitro and in vivo adipogenesis. Diabetes *54*, 402–411.

Brennan, D.F., Dar, A.C., Hertz, N.T., Chao, W.C., Burlingame, A.L., Shokat, K.M., and Barford, D. (2011). A Raf-induced allosteric transition of KSR stimulates phosphorylation of MEK. Nature *472*, 366–369.

Brommage, R., Desai, U., Revelli, J.P., Donoviel, D.B., Fontenot, G.K., Dacosta, C.M., Smith, D.D., Kirkpatrick, L.L., Coker, K.J., Donoviel, M.S., et al. (2008). High-throughput screening of mouse knockout lines identifies true lean and obese phenotypes. Obesity (Silver Spring) *16*, 2362–2367.

Canal, F., Palygin, O., Pankratov, Y., Corrêa, S.A., and Müller, J. (2011). Compartmentalization of the MAPK scaffold protein KSR1 modulates synaptic plasticity in hippocampal neurons. FASEB J. *25*, 2362–2372.

Claret, M., Smith, M.A., Batterham, R.L., Selman, C., Choudhury, A.I., Fryer, L.G., Clements, M., Al-Qassab, H., Heffron, H., Xu, A.W., et al. (2007). AMPK is essential for energy homeostasis regulation and glucose sensing by POMC and AgRP neurons. J. Clin. Invest. *117*, 2325–2336.

Costanzo-Garvey, D.L., Pfluger, P.T., Dougherty, M.K., Stock, J.L., Boehm, M., Chaika, O., Fernandez, M.R., Fisher, K., Kortum, R.L., Hong, E.G., et al.

(2009). KSR2 is an essential regulator of AMP kinase, energy expenditure, and insulin sensitivity. Cell Metab. *10*, 366–378.

Cunningham, J.J. (1991). Body composition as a determinant of energy expenditure: a synthetic review and a proposed general prediction equation. Am. J. Clin. Nutr. *54*, 963–969.

Denouel-Galy, A., Douville, E.M., Warne, P.H., Papin, C., Laugier, D., Calothy, G., Downward, J., and Eychène, A. (1998). Murine Ksr interacts with MEK and inhibits Ras-induced transformation. Curr. Biol. *8*, 46–55.

Dougherty, M.K., Ritt, D.A., Zhou, M., Specht, S.I., Monson, D.M., Veenstra, T.D., and Morrison, D.K. (2009). KSR2 is a calcineurin substrate that promotes ERK cascade activation in response to calcium signals. Mol. Cell *34*, 652–662.

Emsley, P., and Cowtan, K. (2004). Coot: model-building tools for molecular graphics. Acta Crystallogr. D Biol. Crystallogr. *60*, 2126–2132.

Farooqi, S., and O'Rahilly, S. (2006). Genetics of obesity in humans. Endocr. Rev. *27*, 710–718.

Farooqi, I.S., Keogh, J.M., Yeo, G.S., Lank, E.J., Cheetham, T., and O'Rahilly, S. (2003). Clinical spectrum of obesity and mutations in the melanocortin 4 receptor gene. N. Engl. J. Med. *348*, 1085–1095.

Farooqi, I.S., Wangensteen, T., Collins, S., Kimber, W., Matarese, G., Keogh, J.M., Lank, E., Bottomley, B., Lopez-Fernandez, J., Ferraz-Amaro, I., et al. (2007). Clinical and molecular genetic spectrum of congenital deficiency of the leptin receptor. N. Engl. J. Med. *356*, 237–247.

Fernandez, M.R., Henry, M.D., and Lewis, R.E. (2012). Kinase suppressor of Ras 2 (KSR2) regulates tumor cell transformation via AMPK. Mol. Cell. Biol. *32*, 3718–3731.

Greenfield, J.R., Miller, J.W., Keogh, J.M., Henning, E., Satterwhite, J.H., Cameron, G.S., Astruc, B., Mayer, J.P., Brage, S., See, T.C., et al. (2009). Modulation of blood pressure by central melanocortinergic pathways. N. Engl. J. Med. *360*, 44–52.

Guerra, C., Koza, R.A., Walsh, K., Kurtz, D.M., Wood, P.A., and Kozak, L.P. (1998). Abnormal nonshivering thermogenesis in mice with inherited defects of fatty acid oxidation. J. Clin. Invest. *102*, 1724–1731.

Hardie, D.G., Ross, F.A., and Hawley, S.A. (2012). AMPK: a nutrient and energy sensor that maintains energy homeostasis. Nat. Rev. Mol. Cell Biol. *13*, 251–262.

Jager, J., Corcelle, V., Grémeaux, T., Laurent, K., Waget, A., Pagès, G., Binétruy, B., Le Marchand-Brustel, Y., Burcelin, R., Bost, F., and Tanti, J.F. (2011). Deficiency in the extracellular signal-regulated kinase 1 (ERK1) protects leptin-deficient mice from insulin resistance without affecting obesity. Diabetologia *54*, 180–189.

Joneson, T., Fulton, J.A., Volle, D.J., Chaika, O.V., Bar-Sagi, D., and Lewis, R.E. (1998). Kinase suppressor of Ras inhibits the activation of extracellular ligand-regulated (ERK) mitogen-activated protein (MAP) kinase by growth factors, activated Ras, and Ras effectors. J. Biol. Chem. *273*, 7743–7748.

Kola, B., Hubina, E., Tucci, S.A., Kirkham, T.C., Garcia, E.A., Mitchell, S.E., Williams, L.M., Hawley, S.A., Hardie, D.G., Grossman, A.B., and Korbonits, M. (2005). Cannabinoids and ghrelin have both central and peripheral metabolic and cardiac effects via AMP-activated protein kinase. J. Biol. Chem. *280*, 25196–25201.

Kornfeld, K., Hom, D.B., and Horvitz, H.R. (1995). The ksr-1 gene encodes a novel protein kinase involved in Ras-mediated signaling in C. elegans. Cell *83*, 903–913.

Lanctot, A.A., Peng, C.Y., Pawlisz, A.S., Joksimovic, M., and Feng, Y. (2013). Spatially dependent dynamic MAPK modulation by the Nde1-Lis1-Brap complex patterns mammalian CNS. Dev. Cell *25*, 241–255.

Lawrence, M.C., Jivan, A., Shao, C., Duan, L., Goad, D., Zaganjor, E., Osborne, J., McGlynn, K., Stippec, S., Earnest, S., et al. (2008). The roles of MAPKs in disease. Cell Res. *18*, 436–442.

Liu, L., Channavajhala, P.L., Rao, V.R., Moutsatsos, I., Wu, L., Zhang, Y., Lin, L.L., and Qiu, Y. (2009). Proteomic characterization of the dynamic KSR-2 interactome, a signaling scaffold complex in MAPK pathway. Biochim. Biophys. Acta *1794*, 1485–1495.

Lowell, B.B., and Spiegelman, B.M. (2000). Towards a molecular understanding of adaptive thermogenesis. Nature *404*, 652–660.

McKay, M.M., and Morrison, D.K. (2007). Integrating signals from RTKs to ERK/MAPK. Oncogene *26*, 3113–3121.

Minokoshi, Y., Alquier, T., Furukawa, N., Kim, Y.B., Lee, A., Xue, B., Mu, J., Foufelle, F., Ferré, P., Birnbaum, M.J., et al. (2004). AMP-kinase regulates food intake by responding to hormonal and nutrient signals in the hypothalamus. Nature *428*, 569–574.

Mori, H., Inoki, K., Münzberg, H., Opland, D., Faouzi, M., Villanueva, E.C., Ikenoue, T., Kwiatkowski, D., MacDougald, O.A., Myers, M.G., Jr., and Guan, K.L. (2009). Critical role for hypothalamic mTOR activity in energy balance. Cell Metab. *9*, 362–374.

Müller, J., Cacace, A.M., Lyons, W.E., McGill, C.B., and Morrison, D.K. (2000). Identification of B-KSR1, a novel brain-specific isoform of KSR1 that functions in neuronal signaling. Mol. Cell. Biol. *20*, 5529–5539.

Müller, J., Ory, S., Copeland, T., Piwnica-Worms, H., and Morrison, D.K. (2001). C-TAK1 regulates Ras signaling by phosphorylating the MAPK scaffold, KSR1. Mol. Cell *8*, 983–993.

Nguyen, A., Burack, W.R., Stock, J.L., Kortum, R., Chaika, O.V., Afkarian, M., Muller, W.J., Murphy, K.M., Morrison, D.K., Lewis, R.E., et al. (2002). Kinase suppressor of Ras (KSR) is a scaffold which facilitates mitogen-activated protein kinase activation in vivo. Mol. Cell. Biol. *22*, 3035–3045.

Revelli, J.P., Smith, D., Allen, J., Jeter-Jones, S., Shadoan, M.K., Desai, U., Schneider, M., van Sligtenhorst, I., Kirkpatrick, L., Platt, K.A., et al. (2011). Profound obesity secondary to hyperphagia in mice lacking kinase suppressor of ras 2. Obesity (Silver Spring) *19*, 1010–1018.

Roy, F., Laberge, G., Douziech, M., Ferland-McCollough, D., and Therrien, M. (2002). KSR is a scaffold required for activation of the ERK/MAPK module. Genes Dev. *16*, 427–438.

Schuler, A.M., Gower, B.A., Matern, D., Rinaldo, P., Vockley, J., and Wood, P.A. (2005). Synergistic heterozygosity in mice with inherited enzyme deficiencies of mitochondrial fatty acid beta-oxidation. Mol. Genet. Metab. *85*, 7–11.

Shalin, S.C., Hernandez, C.M., Dougherty, M.K., Morrison, D.K., and Sweatt, J.D. (2006). Kinase suppressor of Ras1 compartmentalizes hippocampal signal transduction and subserves synaptic plasticity and memory formation. Neuron *50*, 765–779.

Shaw, R.J. (2009). LKB1 and AMP-activated protein kinase control of mTOR signalling and growth. Acta Physiol. (Oxf.) *196*, 65–80.

Sundaram, M., and Han, M. (1995). The C. elegans ksr-1 gene encodes a novel Raf-related kinase involved in Ras-mediated signal transduction. Cell *83*, 889–901.

Tartaglia, M., and Gelb, B.D. (2010). Disorders of dysregulated signal traffic through the RAS-MAPK pathway: phenotypic spectrum and molecular mechanisms. Ann. N Y Acad. Sci. *1214*, 99–121.

Therrien, M., Chang, H.C., Solomon, N.M., Karim, F.D., Wassarman, D.A., and Rubin, G.M. (1995). KSR, a novel protein kinase required for RAS signal transduction. Cell *83*, 879–888.

Therrien, M., Michaud, N.R., Rubin, G.M., and Morrison, D.K. (1996). KSR modulates signal propagation within the MAPK cascade. Genes Dev. *10*, 2684–2695.

Viollet, B., Guigas, B., Sanz Garcia, N., Leclerc, J., Foretz, M., and Andreelli, F. (2012). Cellular and molecular mechanisms of metformin: an overview. Clin. Sci. *122*, 253–270.

Williams, D.R., Wareham, N.J., Brown, D.C., Byrne, C.D., Clark, P.M., Cox, B.D., Cox, L.J., Day, N.E., Hales, C.N., Palmer, C.R., et al. (1995). Undiagnosed glucose intolerance in the community: the Isle of Ely Diabetes Project. Diabet. Med. *12*, 30–35.

Yang, Y., Atasoy, D., Su, H.H., and Sternson, S.M. (2011). Hunger states switch a flip-flop memory circuit via a synaptic AMPK-dependent positive feedback loop. Cell *146*, 992–1003.

Yu, W., Fantl, W.J., Harrowe, G., and Williams, L.T. (1998). Regulation of the MAP kinase pathway by mammalian Ksr through direct interaction with MEK and ERK. Curr. Biol. *8*, 56–64.

EXTENDED EXPERIMENTAL PROCEDURES

Subjects

The Genetics of Obesity Study (GOOS) is a cohort of 5,000 individuals with severe early-onset obesity; age of obesity onset is less than 10 years. Severe obesity is defined as a body mass index (weight in kilograms divided by the square of the height in meters) standard deviation score greater than 3 (standard deviation scores calculated according to the United Kingdom reference population). 1770 individuals from the cohort of European descent were randomly selected for *KSR2* screening by Sanger sequencing. In addition, we reviewed *KSR2* sequence data obtained from whole-exome sequencing of 331 unrelated individuals from the GOOS cohort.

The Ely study is a prospective population based cohort study of the etiology and pathogenesis of type 2 diabetes and associated conditions in UK Caucasians. 1536 individuals from the cohort were randomly selected for *KSR2* screening.

Genetic Screening

The coding region and intron/exon boundaries of the human *KSR2* gene (ENSG00000171435) were screened in genomic DNA isolated from whole-blood lymphocytes (primers and conditions available on request). Genetic screening was undertaken by PCR, followed by direct sequencing using BigDye terminator chemistry (Applied Biosystems, UK) and analyzed on an ABI 3730 automated sequencer (Applied Biosystems, UK). Exome capture was performed using the Agilent Sure-Select Human All Exon 50 Mb array, followed by high-throughput sequencing using the Illumina HiSeq sequencing system. High-quality sequence data were obtained (mean target coverage of approximately 50× was achieved with 90% of the samples reaching 10× coverage for >80% of target bases). Raw-sequencing reads were mapped to the GRCh37 reference human genome and variants called using Burrows-Wheeler Alignment and Sequence Alignment/Map (SAM) tools. SNPs and insertion/deletions (indels) were identified and realigned using the Genome Analysis Toolkit (GATK).

Statistical Analysis of Rare Variants in Cases versus Controls

We used Fisher's exact test to compare the number of rare (MAF < 0.5%) variants (both SNVs and indels) between cases and controls. Reported results are using the dominant genetic model. Results were robust to using an additive model. Various MAF thresholds were used to define rare variants (0.005, 0.001, 0.0005) with the smallest p value chosen. This process was repeated over 10,000 permutations in order to assess the p value correcting for multiple testing using various MAF thresholds. Finally, the entire process was repeated excluding population controls with a BMI > 30. The analysis was repeated within a replication sample of 238 cases and 1,117 controls and a combined analysis using the original and replication data for a total of 2,339 cases and 2,653 controls was completed. Variants within each MAF category were defined based on the sample for which the test is run (i.e., original, replication, combined).

Generation of KSR2 Expression Constructs

A human cDNA clone encoding the 921 amino acid isoform of KSR2 (ENSEMBL transcript ID: ENST00000425217) was obtained from Origene (SC318393). Standard PCR methods were used to create a clone identical in sequence to the longest 950 amino acid isoform of KSR2 (ENSEMBL transcript ID: ENST00000339824). These were subcloned into the pCR-Blunt vector (Invitrogen) and further subloned as a KpnI/XbaI fragment into pEGFPC1 (N-terminal GFP tag) and pCDNA3.1(+) (N-terminal Flag tag) vectors. Mutagenesis of Flag-KSR2 950 was performed using QuikChange site-directed mutagenesis kit (Stratagene).

Cell Culture and Transfection

HEK293 and Cos7 cells were cultured in DMEM supplemented with 10% fetal bovine serum, 2 mM L-glutamine and 1 mM Pen/Strep. HEK293 cells were transiently transfected with either PEI or Lipofectamine LTX (Invitrogen) for immunoblotting or flow cytometry, respectively. Cos7 cells were transiently transfected with Lipofectamine 2000 (Invitrogen). C2C12 myotubes were cultured in low-glucose DMEM supplemented with 10% fetal bovine serum, 2 mM L-glutamine and 1 mM Pen/Strep and transfected using Lipofectamine LTX Plus (Invitrogen).

Immunoprecipitation and Immunoblotting

24 hr posttransfection, HEK293 cells were serum starved overnight. Cells were then stimulated with 50 ng/ml Human recombinant EGF (Invitrogen) for 10 min. For metformin treatment, transfected HEK293 cells were incubated with 5 mM metformin overnight. Cells were harvested in NP-40 lysis buffer (50 mM Tris pH7.5, 150 mM NaCl, 1 mM EGTA, 1 mM EDTA, 1 mM Sodium orthovanadate, 50 mM Sodium fluoride, 10 mM Sodium pyrophosphate, 10 mM Sodium glycerophosphate, 1% (v/v) NP-40 and protease inhibitors) and lysates were clarified by centrifugation at 12,000 rpm for 15 min at 4°C. Lysates (0.5–1 mg protein) were precleared by incubation with Protein A/G agarose (Santa Cruz Biotechnology) at 4°C for 30 min before incubation with anti-FLAG M2 affinity gel (Sigma) at 4°C for 2 hr. Immunoprecipitates were washed twice with lysis buffer, twice with buffer A (50 mM Tris [pH 7.5], 0.1 mM EGTA), resuspended in SDS sample buffer and filtered through a Spin-X filter to remove the resin. NuPAGE reducing agent (1X; Invitrogen) was added to the eluted samples, which were subjected to electrophoresis and immunoblot analysis. Immunoblotting was performed at 4°C overnight using the following antibodies: anti-Flag (Sigma, #F1804), anti-B-Raf (Santa Cruz Biotechnology, #sc-9002),

anti-ACC [pS79] (#3661], anti-AMPK (#2603), anti-pAMPK [pT172] (#2535), anti-ERK1/2 (#9102), anti-pERK1/2 (#9101), anti-MEK1/2 (#9122), anti-pMEK1/2 [pS217/pS221] (#9121), anti-pRaptor [pS792] (#2083) or anti-pTSC2 [S1387] (Cell Signaling Technology].

Immunofluorescence and Confocal Microscopy

40,000 Cos7 cells were seeded onto glass coverslips in 12-well plates and cultured for 24 hr before transfection using Lipofectamine 2000. After 6 hr, the cells were serum starved overnight. Cells were stimulated with 100 ng/ml EGF for 5 min and then fixed in 4% paraformaldehyde in PBS and subsequently permeabilised with 0.01% Triton X-100 in PBS for 5 min. Coverslips were blocked for 1 hr in 3% BSA in PBS then incubated with the indicated primary antibody. In addition to the antibodies listed above, anti-MEK1 (Santa Cruz Biotechnology, #sc-219) was specifically used for immunostaining. After 1 hr, coverslips were washed in PBS and incubated for 45 min with the appropriate secondary antibody (Dylight488 anti-mouse IgG [Vector Laboratories, DI-2488] or Alexa Fluor568 anti-Rabbit IgG]Molecular probes, A-11036]) before washing in PBS. Coverslips were mounted in Vectashield mounting medium (Vector Laboratories) containing DAPI and examined using a Zeiss LSM510 confocal microscope.

Flow Cytometry

HEK293 cells were seeded into 6-well plates for 24 hr before transfection with 6 μg of Flag-tagged constructs using Lipofectamine LTX. 6 hr posttransfection the cell culture medium was replaced with serum free DMEM. After overnight serum starvation cells were stimulated with 100 ng/ml EGF for 5 min and then harvested using trypsin. Harvested cells were centrifuged and the cell pellet resuspended in 4% formaldehyde in PBS and incubated for 10 min. The following steps were performed in 15 ml falcon tubes: one million cells were permeabilised in staining buffer (3% BSA, 2 mM EDTA and 1% saponin in PBS) for 60 min before immunostaining with primary antibodies (mouse anti-Flag (Sigma, #F1804), and rabbit anti-p-ERK (Cell Signaling Technology, 4370) diluted in staining buffer for 45 min. Cells were rinsed once in washing buffer (1% BSA, 2 mM EDTA and 0.1% saponin in PBS) then stained with secondary antibodies (anti-mouse dylight 488 [Vector Laboratories, DI-2488] and anti-rabbit PE [Molecular probes,A-10542]) diluted in staining buffer for 45 min. Cells were washed once more with washing buffer before resuspension in 700 μl of PBS. Cells were immediately analyzed using a BD FACS Calibur flow cytometer using a 488 nm laser and 530/30 nm filter to detect the anti-mouse daylight 488 secondary antibody signal and 585/42 nm filter to detect the anti-rabbit PE secondary antibody signal. A total of 50,000 events were acquired. Data were interpreted using flowing software 2.

Measurement of Glucose Oxidation

48 hr after transfection, C2C12 cells were subjected to experimental treatment in Seahorse XF24 V7 assay plates coated with poly-L-lysine (20,000 cells/well). Oxygen Consumption Rate (OCR) was then determined using an XF24 extracellular Immuflux analyzer (Seahorse Bioscience) as follows: cells were washed twice in Seahorse assay medium (4.15 g DMEM base, 1.85 g/l NaCl, 1x glutamax, 1 mM sodium pyruvate, 5 mM D-glucose, 15 mg/l phenol red, 20 mM HEPES [pH 7.4]) and incubated in 630 μl of assay medium at 37°C for 1 hr in a non-CO_2 incubator. To investigate cellular response to the presence of respiratory inhibitors and uncouplers 70 μl of oligomycin, 70 μl of FCCP and 70 μl of antimycin A/rotenone were loaded into Seahorse injection ports to achieve a final concentration of 1 μg/ ml oligomycin, 2 μM FCCP, 5 μM antimycin A and 4 μg/ml rotenone. The experimental set-up involved an initial 20 min equilibration step, two cycles of 3 min mix–2 min delay, 3 min measure steps to obtain basal OCR and sequential injections of oligomycin, FCCP and antimycin A/rotenone, each of which was followed by two cycles of mix-delay-measure steps as above.

Oxygen consumption rates (OCR) were normalized to cell number as measured using a sulphorhodamine B (SRB) assay. Cells from Seahorse XF24 extracellular flux experiments were fixed with 200 μl 50% trichloroacetic acid at 4°C for 1 hr. Cells were then washed three times in water and allowed to air-dry. Cells were stained for 20 min at room temperature with 50 μl 0.4% (v/v) SRB in 1% (v/v) acetic acid, and then washed three times in 1% (v/v) acetic acid. The incorporated sulphorhodamine B dye (Sigma) was next solubilised in 100 μl 10 mM unbuffered Tris base at room temperature for 5 min, after which 20 μl of each sample was transferred to fresh wells in duplicates in a 96-well plate and further mixed with 80 μl 10 mM unbuffered Tris base. The absorbance at 565 nm was then measured. To obtain the cell count for each well, the absorbance was compared to a standard curve that was constructed by seeding known number of cells in quadruplicate wells and fixing and staining them in parallel with sample cells.

Measurement of Fatty Acid Oxidation

48 hr posttransfection, C2C12 cells were seeded in Seahorse XF24 V7 assay plates coated with poly-L-lysine (20,000 cells/well) and in order to initiate differentiation, cells were grown for 96 hr in medium with 1% FBS. Cells were either pretreated for 1 hr with 1 mM metformin or left untreated and the Oxygen Consumption Rate (OCR) was then determined using an XF24 extracellular flux analyzer (Seahorse Bioscience) as follows: cells were washed twice in KHB buffer (110 mM NaCl, 4.7 mM KCl, 2 mM $MgSO_4$, 1.2 mM Na_2HPO_4, 2.5 mM glucose adjusted to pH 7.4 and supplemented with 0.5 mM carnitine) and incubated in 630 μl of KHB buffer at 37°C for 1 hr in a non-CO_2 incubator. To investigate FAO to the presence of BSA-conjugated palmitate (Seahorse Bioscience), 70 μl of BSA vehicle, 70 μl of BSA-Palmitate and 70 μl of FAO inhibitor etomoxir (Sigma) were loaded into Seahorse injection ports to achieve final concentration of 33 μM BSA, 100 μM palmitate, 50 μM etomoxir. The experimental set-up involved an initial 20 min equilibration step, two cycles of 3 min mix–2 min delay, 3 min measurement steps to obtain basal OCR and sequential injections of BSA vehicle or BSA-palmitate and etomoxir, each of which was followed by two cycles of mix-delay-measure steps as above. Oxygen consumption rates (OCR) were normalized to cell number as described above.

KSR1 and KSR2 Gene Expression in Human Tissues

A human tissue cDNA library was prepared using 1 μl RNA (Clontech), which was reverse transcribed to cDNA using a Retroscript kit (Ambion). 1 μl cDNA was then used as a template in a PCR reaction using the following primers: KSR1 forward primer, 5′-AAGAG ACTGGCCCTTGAAGAAC-3′; KSR1 reverse primer, 5′-AAGTTTCTCCAGCATGTCCATC-3′; KSR2 forward primer, 5′-GGAGCA AATCCCACGAGTTCCAGC-3′; KSR2 reverse primer, 5′-GGTGCGTGTCCCACAAAGAAGG-3′.

Human Metabolic Phenotyping

Subjects were invited to participate in clinical studies at the Wellcome Trust Clinical Research Facility at Addenbrooke's Hospital, Cambridge, UK. All studies were approved by the Cambridge regional ethics committee and conducted in accordance with the principles of the Declaration of Helsinki. Each subject, or his or her parent for those under 16, provided written informed consent, minors provided oral consent.

Weight and height were measured barefoot in light clothing. Dual X-ray absorptiometry (DEXA) (DPX software; Lunar Corp) was used to determine body composition. Ad libitum energy intake was assessed using a 18MJ breakfast of known macronutrient content after an overnight fast and was expressed per kilogram of lean body mass as measured by DEXA to allow comparison between individuals of different body weights and compositions. Basal metabolic rate and respiratory quotient were determined by indirect calorimetry after an overnight fast using an open circuit, ventilated, canopy measurement system (Europa Gas Exchange Monitor; NutrEn Technology). After adjustment for body composition, basal metabolic rate was compared to predicted metabolic rate based on standard age and sex specific equations. Blood pressure was measured using automated brachial (DINAMAP, GE Healthcare) or wrist (OMRON Healthcare) monitors. Heart rate was recorded using a wearable sensor (Actiheart, CamNtech, Papworth, UK) from which heart rate variability parameters were derived from cleaned interbeat interval time-series. Heart rate was recorded using a wearable sensor (Actiheart, CamNtech, Papworth, UK). Measurements were made during two distinct states; asleep (overnight from 0030–0530 hr); baseline awake (0700 – 0730 hr). Heart rate variability parameters were derived in Kubios HRV software (version 2.1, Kuopio, Finland). Interbeat interval time series were manually cleaned and filtered using the low correction threshold (cleaning interbeat intervals that differ more than 0.35 s from the local mean interbeat interval). Groups were compared using a general linear model after log transformation of RMSSD, low- and high-frequency power (ms2) and LF/HF ratio. Interaction between physiological state (asleep/awake) and groups was also checked in a general linear model (SPSS 20).

Mouse Husbandry and Energy Balance Studies

Ksr2$^{-/-}$ mice, maintained on a mixed genetic background (129/SvEvBrd and C57BL/6J), have been described previously, as have the general methods for mouse husbandry (Brommage et al., 2008; Revelli et al., 2011). The Institutional Animal Care and Use Committee at Lexicon Pharmaceuticals approved all study protocols. All mice were fed a low-fat diet (10% kcal as fat; diet D12450B, Research Diets, New Brunswick, NJ). Pair-feeding studies were performed as described previously (Revelli et al., 2011). Mouse body composition was estimated by Quantitative Magnetic Resonance (QMR, ECHO Medical Systems, Houston, TX) as described previously (Brommage et al., 2008).

Immediately after *Ksr2*$^{-/-}$ mice and WT littermates were housed individually at 4°C, they had their rectal temperature measured for 1 s using a Thermalert TH-8 thermometer (Physitemp Instruments, Clifton, NJ) and Physitemp RET-3 rectal probe. They also had their skin temperature measured by placing a Physitemp MT-4 skin probe over their interscapular space for 1 s. The mice then had their rectal and interscapular skin temperatures re-measured every 30 min over a span of 4 hr. Any mice that became lethargic at a low rectal temperature, or had their rectal temperature drop below 25°C, were removed from the cold room, placed in a warmed cage and their temperature data up to the time of rescue were included in the analyses.

In Situ Hybridization

Twenty μm-thick cryosections of brain were taken from 11-month-old *Ksr2* WT mice. A Ksr2-specific cDNA probe (nucleotides 964-1252, accession number NM_001114545.2) was generated by PCR with primers that incorporate the T7 RNA polymerase promoter sequence into the PCR amplicon. This DNA template was used for in vitro transcription reaction with 8 μCi of α-33P-UTP (NEN Life Science Products, Boston, MA). After hybridization at 60°C for 16hrs, sections were treated with RNase and washed in SSC buffer. Slides were dehydrated in a graded ethanol series and exposed to a 75% solution of autoradiographic emulsion type NTB2 (Eastman Kodak Company, Rochester, NY) for 6 to 10 days. Slides were developed and dehydrated, and coverslips were applied (Permount; Fisher Scientific, Pittsburgh, PA). Digital images were acquired (ORCA II; Hamamatsu, Hamamatsu City, Japan) with a cooled charge-coupled device (CCD) camera mounted on a (BX60; Olympus, Lake Success, NY) microscope equipped with dark-field optics.

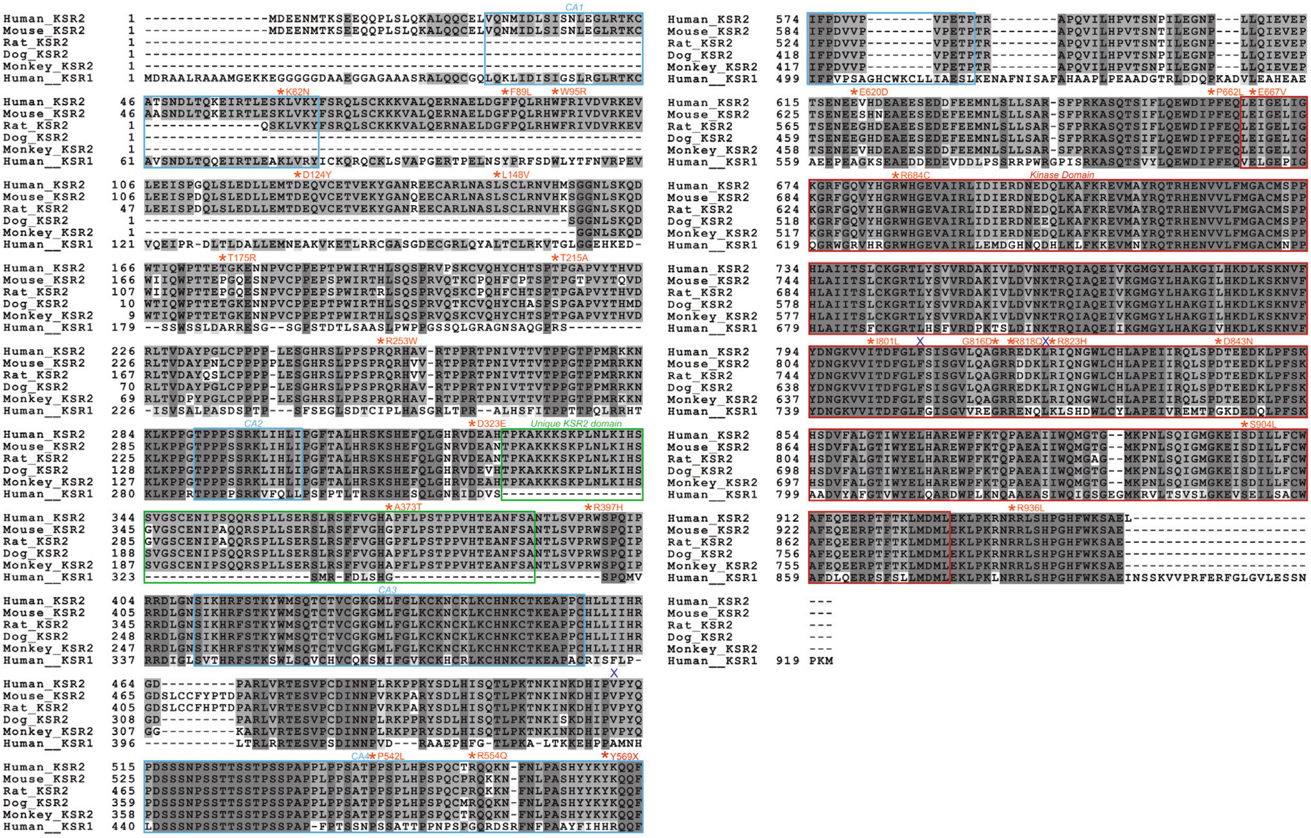

Figure S1. Sequence Alignment of KSR2 Orthologs and KSR1, Related to Figure 1

Sequence alignment of human KSR2 (accession number Q6VAB6), mouse Ksr2 (*Mus musculus* accession number Q3UVC0), Rat Ksr2 (*Rattus norvegicus* accession number FILY04), Dog KSR2 (*Canis familiaris* accession number FIP721), Monkey KSR2 (*Macaca mulatta* accession number F7GRP4) and human KSR1 (accession number Q8IVT5). Sequence alignments were undertaken using a ClustalW alignment program (http://workbench.sdsc.edu/). Dark gray indicates completely conserved residues, mid gray shows partially conserved residues, while light gray indicates similar residues. The KSR2 nonsynonymous missense mutations are indicated in orange, the nonsense mutation Y569X is marked in red and the location of the three frameshift mutations V511Cfsx29, F807Qfsx41 and L822Pfsx26 are shown with a blue X. In addition the CA1-4 domains are shown with a blue box, the domain found only in KSR2 is shown with a green box and the kinase domain is marked with a red box.

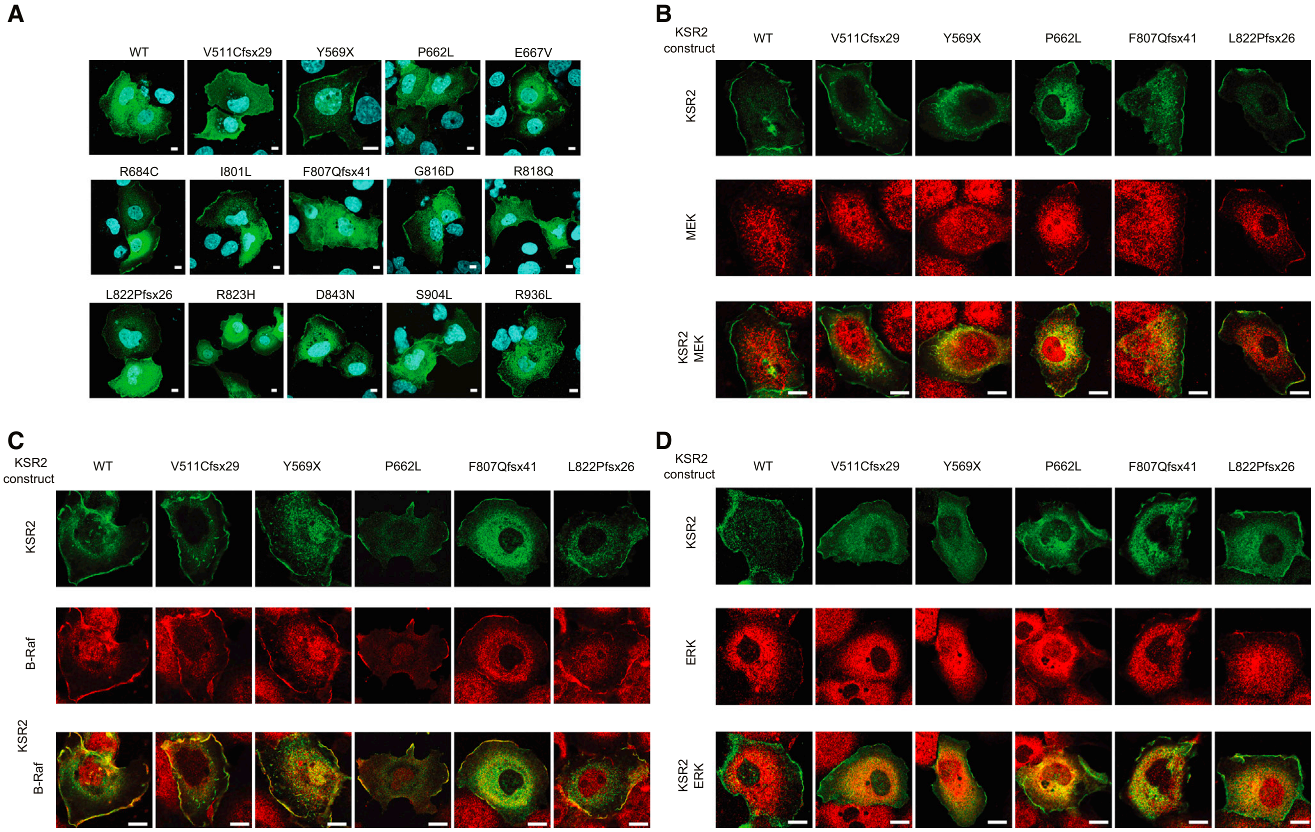

Figure S2. Subcellular Localization of KSR2 Mutants and Colocalization with B-Raf, MEK, and ERK, Related to Figure 2

(A) Serum starved Cos7 cells transfected with Flag tagged WT or mutant KSR2 were stimulated with 100 ng/ml EGF for 5 min and fixed before immunostaining with an anti-Flag antibody (green) and DNA staining with DAPI (blue). Confocal optical sections were chosen that show KSR2 cellular localization in both the cytoplasm and plasma membrane ruffles. Scale bars, 10 μm.

(B–D) Serum starved Cos7 cells transfected with Flag tagged WT or mutant KSR2 were stimulated with 100 ng/ml EGF for 5 min and fixed. Cells were coimmunostained with anti-Flag (green) and either: anti-B-Raf, anti-MEK or anti-ERK (red) antibodies and analyzed by confocal microscopy. Scale bars, 10 μm.

```
WT           500  KTNKINKDHIPVPYQPDSSSNPSSTTSSTPSSPAPPLPPSATPPSPLHPSPQCTRQQKNF
V511Cfsx29   500  KTNKINKDHIPCPLPARLQQQPLLHDVLHALLASTPPPS---------------------
Y569X        500  KTNKINKDHIPVPYQPDSSSNPSSTTSSTPSSPAPPLPPSATPPSPLHPSPQCTRQQKNF
F807Qfsx41   500  KTNKINKDHIPVPYQPDSSSNPSSTTSSTPSSPAPPLPPSATPPSPLHPSPQCTRQQKNF
L822Pfsx26   500  KTNKINKDHIPVPYQPDSSSNPSSTTSSTPSSPAPPLPPSATPPSPLHPSPQCTRQQKNF
                  ERK binding
WT           560  NLPASHYYKYKQQFIFPDVVPVPETPTRAPQVILHPVTSNPILEGNPLLQIEVEPTSENE
V511Cfsx29        ------------------------------------------------------------
Y569X        560  NLPASHYYK---------------------------------------------------
F807Qfsx41   560  NLPASHYYKYKQQFIFPDVVPVPETPTRAPQVILHPVTSNPILEGNPLLQIEVEPTSENE
L822Pfsx26   560  NLPASHYYKYKQQFIFPDVVPVPETPTRAPQVILHPVTSNPILEGNPLLQIEVEPTSENE
                                                                     β1
WT           620  EVHDEAEESEDDFEEMNLSLLSARSFPRKASQTSIFLQEWDIPFEQLEIGELIGKGRFGQ
V511Cfsx29        ------------------------------------------------------------
Y569X             ------------------------------------------------------------
F807Qfsx41   620  EVHDEAEESEDDFEEMNLSLLSARSFPRKASQTSIFLQEWDIPFEQLEIGELIGKGRFGQ
L822Pfsx26   620  EVHDEAEESEDDFEEMNLSLLSARSFPRKASQTSIFLQEWDIPFEQLEIGELIGKGRFGQ
                    β2       β3                αC                    β4      β5
WT           680  VYHGRWHGEVAIRLIDIERDNEDQLKAFKREVMAYRQTRHENVVLFMGACMSPPHLAIIT
V511Cfsx29        ------------------------------------------------------------
Y569X             ------------------------------------------------------------
F807Qfsx41   680  VYHGRWHGEVAIRLIDIERDNEDQLKAFKREVMAYRQTRHENVVLFMGACMSPPHLAIIT
L822Pfsx26   680  VYHGRWHGEVAIRLIDIERDNEDQLKAFKREVMAYRQTRHENVVLFMGACMSPPHLAIIT
                           αD                  αE                      β7
WT           740  SLCKGRTLYSVVRDAKIVLDVNKTRQIAQEIVKGMGYLHAKGILHKDLKSKNVFYDNGKV
V511Cfsx29        ------------------------------------------------------------
Y569X             ------------------------------------------------------------
F807Qfsx41   740  SLCKGRTLYSVVRDAKIVLDVNKTRQIAQEIVKGMGYLHAKGILHKDLKSKNVFYDNGKV
L822Pfsx26   740  SLCKGRTLYSVVRDAKIVLDVNKTRQIAQEIVKGMGYLHAKGILHKDLKSKNVFYDNGKV
                  β8         Activation Segment          αEF                 αF
WT           800  VITDFGLFSISGVLQAGRREDKLRIQNGWLCHLAPEIIRQLSPDTEEDKLPFSKHSDVFA
V511Cfsx29        ------------------------------------------------------------
Y569X             ------------------------------------------------------------
F807Qfsx41   800  VITDFGLQHFWGAAGWQAGGQTAHPEWLAMPPGTRDHPPAVPRHRGG-------------
L822Pfsx26   800  VITDFGLFSISGVLQAGRREDKPHPEWLAMPPGTRDHPPAVPRHRGG-------------
                                         αG                     αH
WT           860  LGTIWYELHAREWPFKTQPAEAIIWQMGTGMKPNLSQIGMGKEISDILLFCWAFEQEERP
V511Cfsx29        ------------------------------------------------------------
Y569X             ------------------------------------------------------------
F807Qfsx41        ------------------------------------------------------------
L822Pfsx26        ------------------------------------------------------------

WT           920  TFTKLMDMLEKLPKRNRRLSHPGHFWKSAEL
V511Cfsx29        -------------------------------
Y569X             -------------------------------
F807Qfsx41        -------------------------------
L822Pfsx26        -------------------------------
```

Figure S3. Effect of Frameshift Mutations and Nonsense Mutation upon the KSR2 Kinase Domain, Related to Figure 3

Amino acid sequences of wild-type KSR2 (500-end) are shown together with the truncated forms of KSR2 that result from each of the V511Cfsx29, F807Qfsx41 and L822Pfsx26 mutations. Sequence alignments were undertaken using http://workbench.sdsc.edu/. The ERK-binding motif is indicated along with key features of the KSR2 kinase domain.

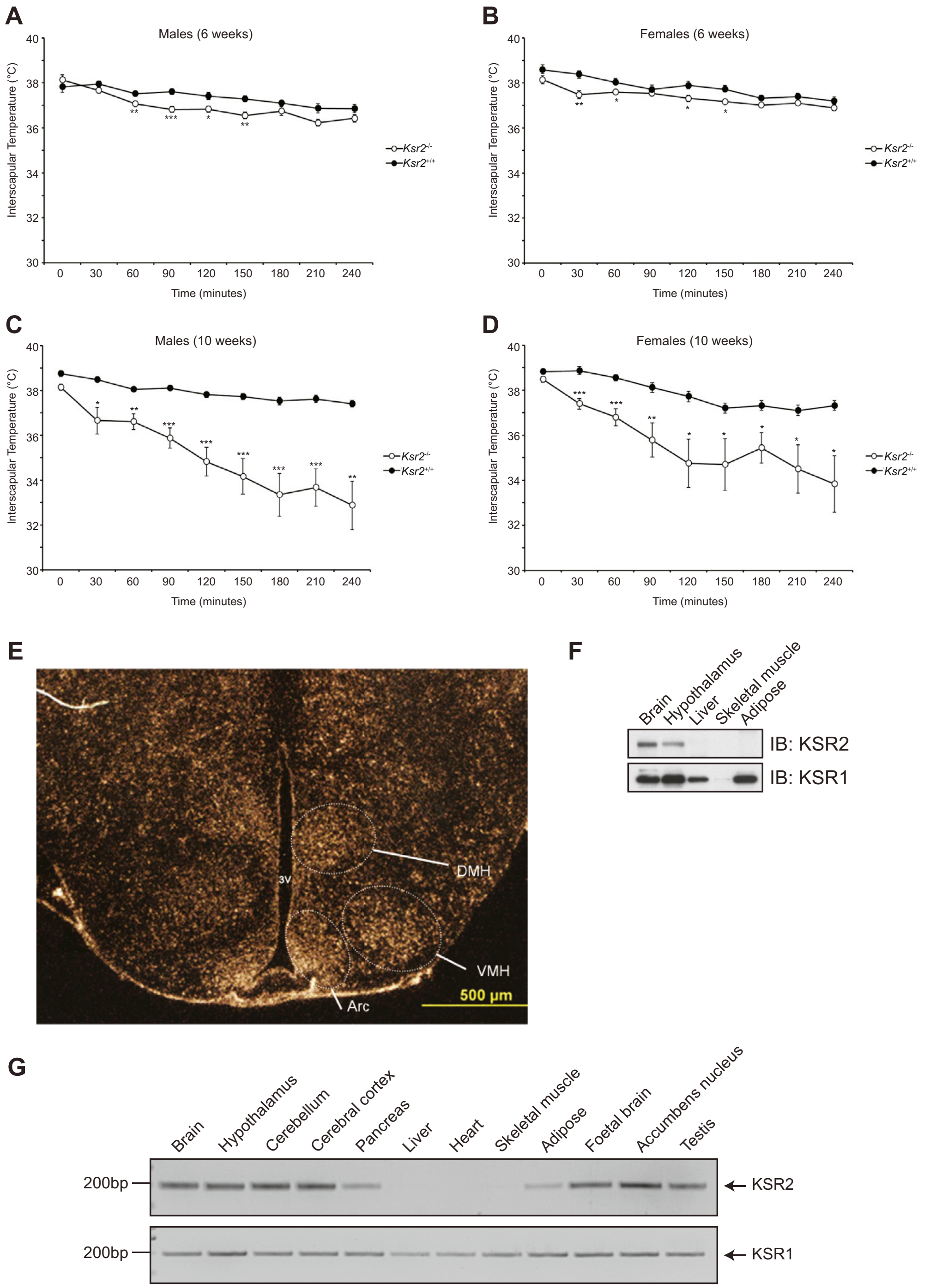

Figure S4. Impaired Cold Tolerance in *Ksr2* KO Mice and KSR2 Expression Pattern, Related to Figure 5

(A) Interscapular temperatures of 6 week-old male $Ksr2^{+/+}$ (n = 9) and $Ksr2^{-/-}$ (n = 10) mice following cold exposure at 4°C.

(B) Interscapular temperatures of 6 week-old female $Ksr2^{+/+}$ (n = 10) and $Ksr2^{-/-}$ (n = 9) mice following cold exposure at 4°C.

(C) As in A) except measurements were made on the same male mice at 10 weeks of age.

(D) As in (B) except measurements were made on the same female mice at 10 weeks of age. Different from WT: *$p < 0.05$, **$p < 0.01$, ***$p < 0.001$.

(E) Ksr2 expression in mouse hypothalamus by in situ hybridization. Arc, arcuate nucleus; DMH, dorsomedial hypothalamus; VMH, ventromedial hypothalamus.

(F) Tissues isolated from fed wild-type mice were lysed and subjected to immunoblotting with the indicated antibodies.

(G) Expression of KSR2 and KSR1 were studied by qualitative RT-PCR in a panel of human tissues.

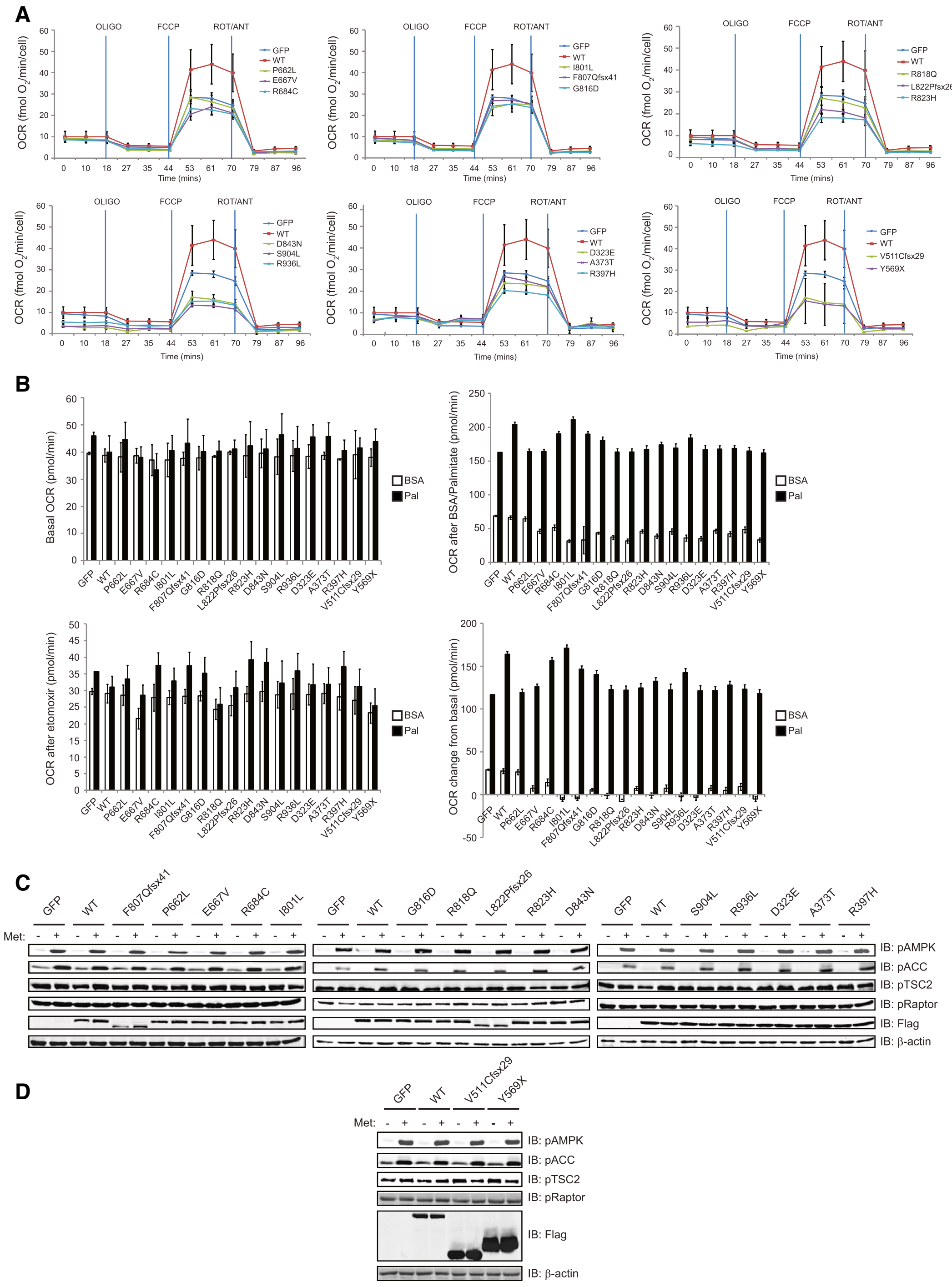

Figure S5. Effects of Human *KSR2* Mutations upon Glucose and Fatty Acid Oxidation, Related to Figure 6

(A) Measurement of glucose oxidation in intact C2C12 cells by Seahorse XF24 extracellular flux analyzer. The graphs show OCR versus time in the presence of 1 μg/ml oligomycin (OLIGO), 2 μM FCCP and 4 μg/ml rotenone/5 μM antimycin (ROT/ANT).

(B) Measurement of FAO in differentiated C2C12 cells overexpressing WT and mutant forms of KSR2 by the Seahorse XF24 extracellular flux analyzer. Top left: basal oxygen consumption rate (OCR), top right: OCR after injection of 33 μM BSA or 100 μM palmitate (Pal), bottom left: OCR after injection of 50 μM etomoxir, bottom right: OCR change from basal.

(C and D) Lysates from C2C12 cells transfected with the indicated KSR2 constructs were treated for 1 hr with 1 mM metformin and immunoblotted with the indicated antibodies.
